# Patterns of association and distribution of estuarine-resident common bottlenose dolphins *(Tursiops truncatus*) in North Carolina, USA

**DOI:** 10.1371/journal.pone.0270057

**Published:** 2022-08-15

**Authors:** Aleta A. Hohn, Antoinette M. Gorgone, Barbie L. Byrd, Kyle W. Shertzer, Tomo Eguchi

**Affiliations:** 1 Cooperative Institute for Marine and Atmospheric Studies, Rosenstiel School for Marine and Atmospheric Science, University of Miami, Miami, Florida, United States of America, at the NOAA Beaufort Laboratory, Beaufort, North Carolina, United States of America; 2 Protected Resources Program North Carolina Division of Marine Fisheries North Carolina Department of Environmental Quality, Morehead City, North Carolina, United States of America; 3 Southeast Fisheries Science Center, National Marine Fisheries Service, National Oceanic and Atmospheric Administration, Beaufort, North Carolina, United States of America; 4 Southwest Fisheries Science Center, National Marine Fisheries Service, National Oceanic and Atmospheric Administration, La Jolla, California, United States of America; Hawaii Pacific University, UNITED STATES

## Abstract

The social structure of estuarine-resident bottlenose dolphins is complex and varied. Residing in habitats often utilized for resource exploitation, dolphins are at risk due to anthropogenic pressures while still federally protected. Effective conservation is predicated upon accurate abundance estimates. In North Carolina, two estuarine-resident stocks (demographically independent groups) of common bottlenose dolphin have been designated using spatiotemporal criteria. Both stocks are subjected to bycatch in fishing gear. The southern North Carolina estuarine stock was estimated at <200 individuals from surveys in 2006, which is outdated per US guidelines. Thus, we conducted a new capture-mark-recapture survey in 2018, identifying 547 distinct individuals, about three times higher than the prior abundance estimate. We compared those individuals to our long-term photo-identification catalog (1995–2018, n = 2,423 individuals), matching 228 individuals. Of those 228, 65 were also included in the 2013 abundance estimate for the northern North Carolina estuarine stock. Using sighting histories for all individuals in the long-term catalog, we conducted a social network analysis, which is independent of *a priori* stock assignments. The three primary clusters identified were inconsistent with current stock designations and not defined by spatiotemporal distribution. All three clusters had sighting histories in the estuary and on the coast, however, that with the highest within-cluster associations appeared to use estuarine waters more often. The within-cluster association strength was low for one cluster, possibly due to only part of that cluster inhabiting the southern North Carolina estuarine system. Between-cluster differences occurred in infestation rates by the pseudostalked barnacle, *Xenobalanus globicipitis*, but that did not predict clusters. We suggest the need to re-evaluate the stock structure of estuarine-resident common bottlenose dolphins in North Carolina and currently have insufficient information to assign an abundance estimate to a currently designated stock.

## Introduction

The social and stock structure of bottlenose dolphins, *Tursiops* spp., is demonstrably complex and varied [1–3]. Numerous studies have identified fine-scale structure using sighting histories, group and habitat characteristics, telemetry, genetics, and, increasingly, from social-network analyses, often resulting in groups comprising <100 individuals [4–14]. In estuarine and nearshore habitats, dolphins are exposed to numerous anthropogenic threats, such as interactions with and bycatch in fishing gear, vessel strikes, provisioning, and acoustic disruption [e.g., 8, 15–20], and environmental contaminants that are sufficiently severe as to cause demographic effects [e.g., 21–24]. As a result, it has been suggested that management and conservation measures be applied at an appropriate spatial scale for the fine-scale population structure [7, 25].

In the United States (US), management of marine mammals is by designated stock. As defined in the US Marine Mammal Protection Act (MMPA), a stock is “a group of marine mammals of the same species or smaller taxa in a common spatial arrangement that interbreed when mature” with a goal that the abundance is sufficient such that the stock remains a functioning component of the ecosystem (https://www.ecfr.gov/current/title-50/chapter-I/subchapter-B/part-18). Current interpretation of the MMPA recognizes a stock as “a management unit that is a demographically independent population (DIP)” wherein the primary influences on its population dynamics are births and deaths rather than immigration or emigration [26]. While a designated stock generally comprises one DIP, it is possible that multiple DIPs could be designated as a single stock for management [26].

The currently understood stock structure of common bottlenose dolphins (*Tursiops truncatus*, hereafter bottlenose dolphins) along the Atlantic coast of the United States (US) is complex, including resident, transient and migratory individuals in both coastal and estuarine habitats [27]. Residents use an area almost exclusively, while transients transit among residents within a site but do not remain for long periods of time [28–32]. Such diversity probably reflects the complexity of the numerous bays, sounds, and estuaries, and a wide, gently sloping (average < 1°) [33] continental shelf used by both coastal and estuarine dolphins. Along the Atlantic coast, 10 such stocks, including known or putative resident stocks in each of the bays, sounds, and estuaries, have been designated [27, 34]. While genetic differentiation is not required for stock designation, differences have been found between a limited number of estuarine and coastal stocks and between groups within estuaries [35, 36], with supporting data from radio telemetry and stable isotope ratios [27]. Also, it has been suggested that the presence of the monospecific commensal barnacle, *Xenobalanus globicipitis* (hereafter *Xenobalanus*) [37], may be used to distinguish between sympatric coastal and estuarine-resident dolphins [36, 38]. Currently, however, stock designation primarily is a function of spatiotemporal distribution, documented principally from summer surveys using resightings of identified individuals [27].

**Fig 1:**
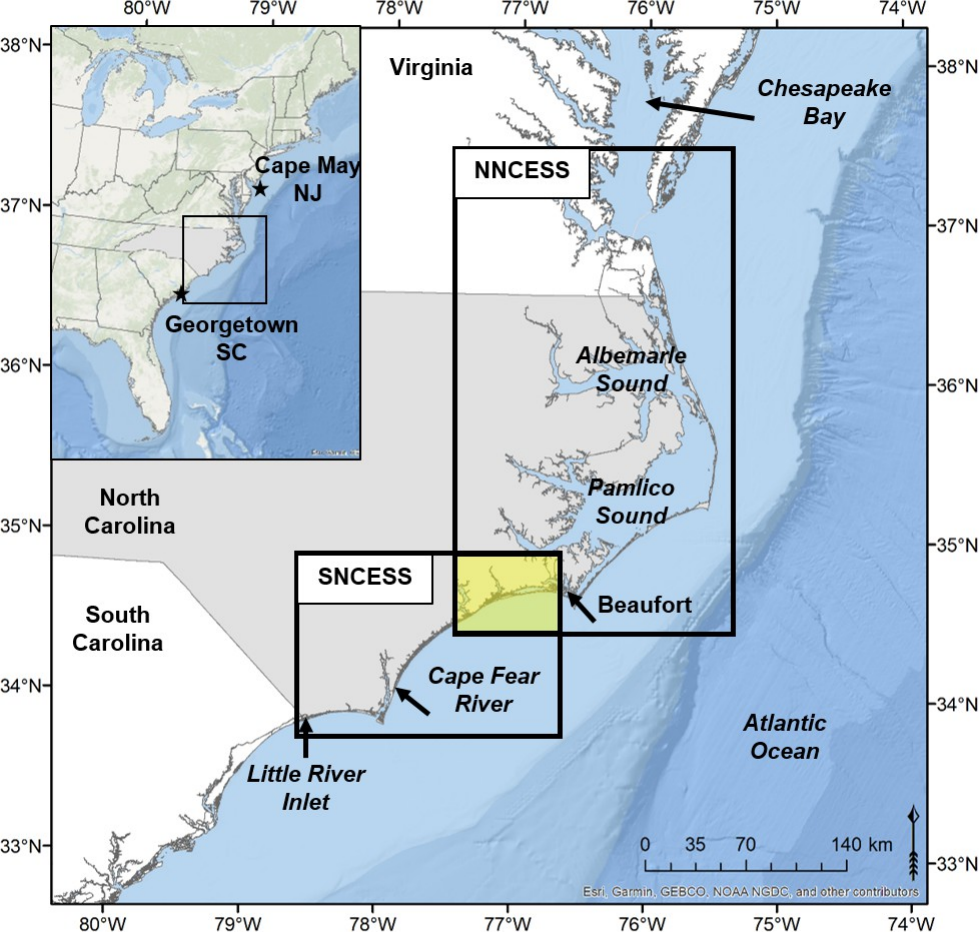
Geography of the North Carolina coast and stock boundaries for the two designated estuarine-resident bottlenose dolphin stocks. The two boxes labelled NNCESS (Northern North Carolina Estuarine System Stock) and SNCESS (Southern North Carolina Estuarine System Stock) indicate the currently defined range, across all seasons, of the two estuarine-resident stocks of bottlenose dolphins in North Carolina. The yellow-shaded area indicates an area of overlap for the two stocks. During some months, the stock definitions have the two stocks sympatric in this area. The long-term photo-ID catalog contains identified dolphins photographed from Cape May, NJ, to Georgetown, SC.

Four stocks of bottlenose dolphin are recognized in estuarine and near-shore coastal waters of North Carolina (NC), US [27] (Fig 1; basemap was generated from ArcGIS by ESRI “Ocean Basemap”, scale not given, accessed April 8, 2022, https://www.arcgis.com/home/item.html?id=6348e67824504fc9a62976434bf0d8d5, from sources ESRI, GEBCO, NOAA, National Geographic, DeLorme, Geonames.org, and other contributors). Two stocks are resident in the NC estuarine system, the northernmost estuarine system along the Atlantic coast of the US with resident bottlenose dolphins; two stocks are migratory [27]. Currently, the Northern NC Estuarine System Stock (NNCESS) is defined as primarily inhabiting northern and central estuarine waters of NC with forays of individuals into nearshore (<3 km) coastal waters [27] and into coastal waters north of NC during warm-water months; individuals are found in the NC central estuary year-round [39]. The Southern NC Estuarine System Stock (SNCESS) is defined as inhabiting southern NC estuarine and nearshore coastal waters during cold-water months with individuals moving into central NC estuarine waters in warm-water months, where they are sympatric with the NNCESS [34]. The two estuarine stocks are also seasonally sympatric with the two coastal stocks, the Northern Migratory Coastal Stock and Southern Migratory Coastal Stock, in nearshore coastal waters [27].

As with many of the estuarine-resident stocks along the US Atlantic and Gulf of Mexico coasts, the population sizes for the two NC estuarine stocks are small with resulting small annual thresholds for sustainable human-caused mortalities (Potential Biological Removal level [PBR]) [27] (S1 File). The NNCESS has been estimated to be <1000 individuals, while a now outdated estimate for SNCESS suggested an abundance of <200 individuals [40]. The difference in abundance between the two stocks resulted in different PBR levels [26], 1.6 animals and 7.8 individuals annually for SNCESS and NNCESS, respectively [27, 41]. Current abundance estimates and resulting PBR levels for these small stocks in NC are important to determine due to their risk of incidental mortality (i.e., bycatch) or serious injury in commercial fisheries [42–45]. Further, observer coverage is low and for SNCESS bycatch information is primarily from stranded animals or fisher reports [42–44]. The current bycatch of SNCESS could be a minimum of two animals, although assigning observed bycaught dolphins to stock is confounded by the four overlapping stocks in coastal waters (S1 File).

We undertook a survey within the southern NC estuarine system in 2018 to provide an updated estimate of abundance for the SNCESS using capture-mark-recapture (CMR) techniques. The survey was strategically planned for winter when little overlap in distribution with the NNCESS or coastal migratory stocks was expected [34]. Because sighting histories have been an important tool for identifying or confirming stock membership, we also compared dolphins that were photo-identified during the survey to dolphins that were included in a long-term photo-identification (ID) catalog. Given unexpected results, using the long-term catalog we conducted a social network analysis as a means of identifying social groups without using *a priori* spatiotemporal strata to see whether associations among individuals were consistent with current stock designations. Using the same datasets (*i*.*e*., 2018 survey and the long-term photo-ID catalog), we also evaluated the use of differences in *Xenobalanus* infestation among individuals and groups as support for stock definition. Furthermore, as sea surface temperatures during the survey were lower than average and the number of groups and dolphins sighted were higher than expected, we examined whether anomalously low water temperatures may have resulted in movement of NNCESS dolphins into more southern waters (S2 File) during the survey. An estimate of abundance was obtained for the survey area although the interpretation was confounded by sighting histories and association patterns that were inconsistent with the currently designated stock structure. Our findings suggest the need to reevaluate stock structure of estuarine-resident bottlenose dolphins in North Carolina, which may be facilitated by use of long-term association patterns.

## Materials and methods

### Ethics statement

Research was conducted under National Marine Fisheries Service Scientific Research Permit 779–1633. All research protocols used were approved by a NOAA Institutional Animal Care and Use Committee.

### Description of habitat

Both NC estuarine stocks inhabit the second largest estuarine complex in the US [33]. It is bounded on the east by barrier islands, separated by numerous inlets, and on the west by the continental US or inflowing rivers (Fig 1). The largest expanses of water (5,300 km^2^) are contained within the Pamlico-Albemarle complex, a lagoon estuary system, which occurs in the central and northern areas; mean depth is 4.5m [46–48]. This shallow water is no impediment to dolphin movements as evidenced by dolphins commonly seen in waters < 2m deep during aerial surveys over the southern portion of the Pamlico-Albemarle complex [39]. South of the Pamlico-Ablemarle complex, the estuarine system includes the Cape Fear River estuary and a series of narrow corridors primarily along the inland navigable channel (Intracoastal Waterway [ICW]) and associated small rivers, tributaries, and channels. For example, the width of the ICW corridor throughout most of the southern area is between 0.1 and 0.2 km, inclusive of the shallow non-navigable edges. With about 22 inlets and the ICW, there is no barrier to movements of dolphins within and between the estuarine system and adjacent coastal waters.

### Surveys and data collection

**Fig 2:**
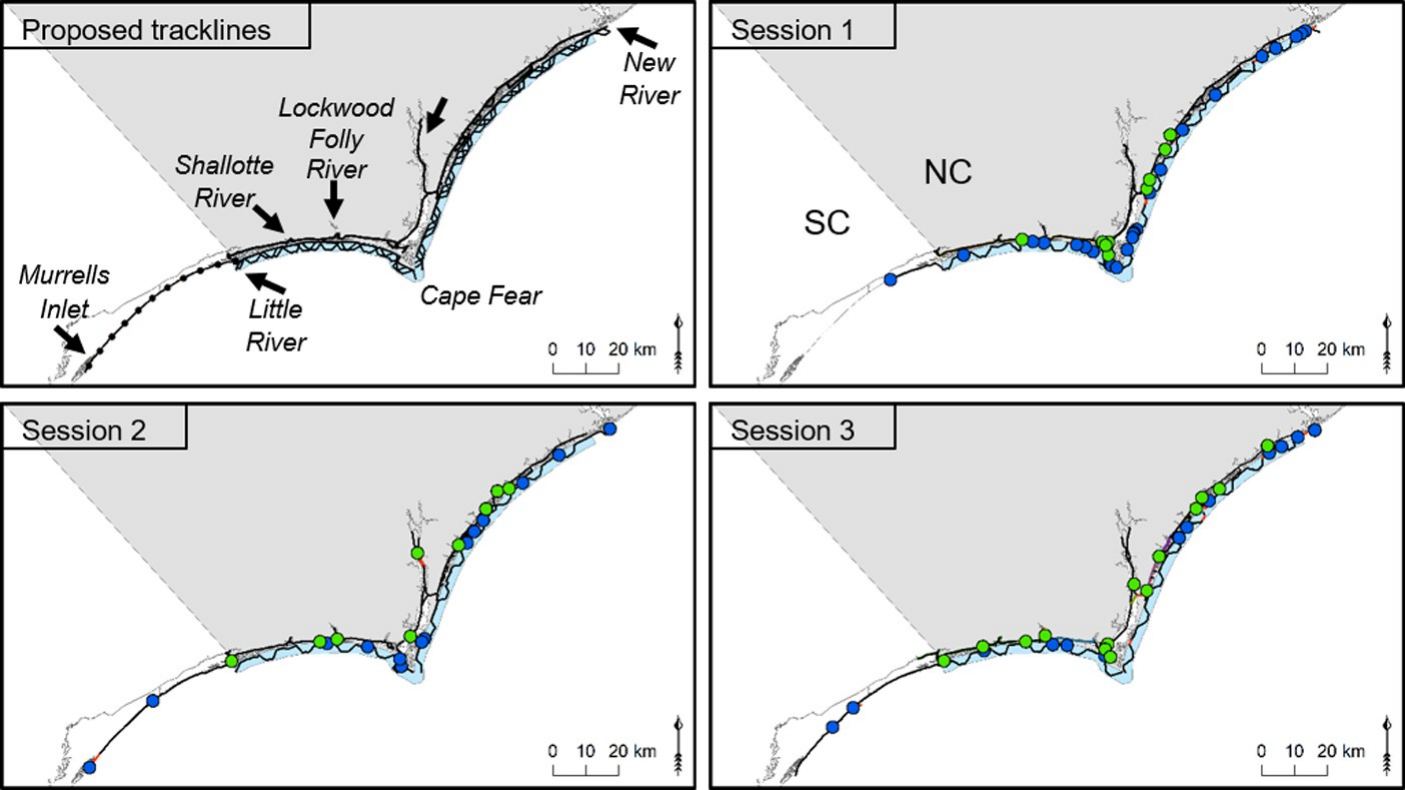
Survey tracklines and resulting sightings. The January 2018 capture-mark-recapture survey for abundance estimates of common bottlenose dolphins (*Tursiops truncatus*) of the designated Southern North Carolina Estuarine System Stock was designed with three photo-ID sessions. The proposed tracklines indicate the survey design, with the coastal tracklines offset so that none of the sessions had the same starting point. The actual tracklines and sightings along those tracking are shown for each of the three sessions. Habitat of sightings is indicated by color—blue circles indicate sightings on the coast and green circles indicate sightings in the estuary.

Surveys were conducted during 8–26 January 2018. Following Rosel et al. [49] and for consistency with the most recent NNCESS abundance estimate [50], we employed three sampling sessions within a limited time frame during which no demographic changes would be expected [51]. The surveys occurred in estuarine and coastal waters corresponding to the defined winter boundary of SNCESS, *i*.*e*., from just south of New River Inlet to Little River Inlet at the NC/South Carolina (SC) border within the estuarine system and in coastal waters to 3 km from shore [34] (Fig 2). In addition, surveys in coastal waters were extended north to include the 5.5 km linear distance between the SNCESS boundary and New River Inlet (hereafter NRI_Trackline), from which the survey vessel entered coastal waters. Surveys also extended 57.4 linear km south from Little River Inlet to Murrells Inlet, SC, (hereafter SC_Trackline) to cover an area of uncertainty with regard to the southern distribution of SNCESS [27, 52]. Tracklines were created in ArcMap (v 10.5, ESRI, Redlands, CA). Along the NC coast, tracklines followed a zig-zag pattern comprising a series of 6 km nearshore transects, where estuarine dolphins are more likely to occur, with parallel 2 km transects offshore, and the connecting diagonal. Because visual detection of dolphin groups can occur up to approximately 500 m from the boat, the nearshore sections were 500 m from shore allowing for full coverage out to 1 km from shore [40]. Similarly, offshore sections extended to 2.5 km allowing for coverage out to 3 km from shore, per the defined SNCESS habitat in winter. Starting points were chosen randomly for each session and were offset from prior starting points (Figs 2 and S1). Within the estuary, tracklines were predetermined for the ICW and the Cape Fear, Shallotte, and Lockwood Folly Rivers, but exact lines were governed by the narrow waterways and shallow water. The estuarine system in most of the area surveyed is only between 0.1 and 0.2 km wide behind narrow barrier islands. Thus, all accessible areas were surveyed including small creeks and boat channels. For coastal and estuarine waters, predetermined tracks were adjusted, as required, due to areas inaccessible at low tide or to shoaling, especially around creeks and inlets. The resulting planned coastal and estuarine tracklines covered approximately 493 km linear distance (235 km coastal, 258 km estuary).

The surveys were conducted using two boats to maximize coverage during each good-weather day given the short day-lengths in winter. During each session, we anticipated five days of effort with one boat surveying along the coast and the other simultaneously surveying in the estuary. As time allowed, exploratory coverage for opportunistic photo-ID sampling of dolphins was conducted in estuarine waters of SC from just south of the winter boundary of the SNCESS, and in the New River estuary, through which the vessels accessed New River Inlet. Photos from the opportunistic sampling, however, were not included in, the abundance estimation process.

Survey methods followed the same protocol on each boat. Personnel included a boat driver, primary photographer, and data recorder/secondary photographer; the same three individuals on each boat participated in all surveys. All three individuals served as observers, scanning from 90° port to 90° starboard, while surveying. Surveys occurred in Beaufort sea-states of 3 or less and at a boat speed of 18–28 km/hr. Geographic coordinates, sea-surface temperature (SST), and depth were collected every 30 seconds from each boat using a Garmin echoMAP GPS with Garmin GT21-TM Transom Mount transducer or a Garmin GPSMAP 942xs with Garmin GT23M-TM Transom Mount Transducer using mid-band CHIRP traditional sonar. When a dolphin group was sighted, survey effort was suspended (off-effort) and the group was approached for photo-sampling. Groups were defined as animals within five body lengths using the chain rule [53]. Photographs of dorsal fins primarily were taken with either Canon 7D Mark II or 7D, both with 100–400 mm lenses. Secondary photographers used a Canon EOS 40D camera with 100–300 mm lens. Attempts were made to photograph all animals in each group regardless of fin distinctiveness or whether a dolphin had previously been photographed to minimize bias due to photographing only well-marked individuals [54]. Encounters ended when all dolphins had been photographed. The exception was in coastal waters; sampling did not exceed 30 minutes if the group likely comprised coastal migratory dolphins (see below) because these animals would not be included in the abundance estimate. For each dolphin group sighted, geographic coordinates and time were recorded when in close proximity to the group rather than from the potentially distant location when the dolphin group was first seen. At the end of each encounter, data were recorded for group size, depth, SST and salinity. SST and salinity were measured using a portable (YSI) meter. Additionally, the presence of *Xenobalanus* was recorded as the relative number of *Xenobalanus* on dorsal fins of the group (infestation load -none, low, medium, or heavy) and the percentage of the group with the barnacle [55]. Groups were assumed to be coastal migratory if (1) the group infestation load was >70% and individual dolphins had an infestation load of medium or heavy [55] and (2) the animals exhibited avoidance behavior such that they were not easily approachable for photo-sampling [13]. At the conclusion of an encounter, the vessel returned to the trackline and survey effort resumed.

For groups identified as estuarine resident, we tested for differences in environmental parameters and group size between groups sighted in the estuary and on the coast (t-tests).

### Photo-identification processing

After all surveys were completed, photographs were reviewed to identify individual dolphins from the presence, size, and location of nicks and notches on the dorsal fin [56]. The best photograph for each individual dolphin within the encounter was selected and assigned a distinctiveness rating (not distinctive, slightly distinctive, distinctive, or very distinctive), and a photo quality rating (poor, fair, good, or excellent) based on focus, size of the fin relative to the photo frame, and angle of the dorsal fin relative to the photo frame [5]. Photographs with a quality rating of poor were excluded from further consideration. A combination of distinctiveness and photo quality determines whether a photograph can be used for long-term photo-ID from long-term marks (hereafter LTM) (S3 File). Advances in high resolution DSLR cameras can allow for ID of individuals using features associated with lower distinctiveness (*e*.*g*., small notches, dorsal fin shape, color variation) or short-term marks such as tooth rakes, scars, and associations (e.g., mom/calf pairs), collectively referred to as short-term marks (STM) (S3 File) [5, 57]. Following Gorgone et al. [50] we assumed that unique features would not change during a short period of time and included both sets of data (LTM and STM) in the current analysis. While calves are often unmarked, we encountered only seven during the survey and each was marked. Thus, they were included as marked animals.

A photo-ID catalog of individuals from the 2018 SNCESS survey was created (2018 SNCESS Catalog), excluding coastal migratory groups. Two experienced researchers (one of whom was always co-author AMG) reviewed the catalog for and agreed upon matches. When possible, matches were made using the left and right sides of the individual. Computer-assisted matching using FinFindR (https://github.com/haimeh/finFindR) was then conducted to review the catalog again for missed matches. FinFindR is an open source R package that uses an edge-tracing algorithm for the trailing edge of the dorsal fin and a matching algorithm to compare the traces of dorsal fins within and between catalogs [58]. As a result of the speed of FinFindR, photos from individually identified dolphins from coastal migratory groups were also compared to individuals from the SNCESS groups, although only a portion of the coastal migratory dolphins could be identified due to the heavy loads of *Xenobalanus* obscuring dorsal-fin features.

Individually identified dolphins from the 2018 SNCESS Catalog were also compared, using FinFindR, to a long-term photo-ID catalog (hereafter NOAA Beaufort Catalog) archived at the NOAA Beaufort Laboratory in NC. The NOAA Beaufort Catalog includes photographs of 2,423 individual dolphins from Cape May, New Jersey, to Georgetown, SC, (Fig 1) from 1995–2018. The images from 1995 were from a capture-release survey. The majority of photos were taken during systematic surveys and daily, local surveys conducted from 2001 to 2018. The surveys comprised 614 vessel days, 50% of which occurred during summer while the remainder were evenly divided among the other three seasons. Spatially, the systematic surveys included photo-ID surveys from Beaufort, NC, to Georgetown, SC, summer 2003, and coastal waters off Beaufort, NC, in 2016; biopsy surveys of the NNCESS in 2010 [59], CMR surveys of the NNCESS in 2013 and biopsy surveys of the SNCESS in late 2014 and early 2015 (Fig 1). Thus, spatial coverage was primarily in North Carolina waters and included and exceeded the anticipated range of the SNCESS.

### 2018 sightings relative to designated stocks

Sightings histories for individuals in the 2018 SNCESS Catalog were reviewed for spatial and temporal patterns relative to stocks boundaries for the SNCESS and NNCESS. In summary, the patterns are defined using 16 geographic strata within 2-month intervals based on the best available knowledge of movements of the stocks [45]. For the current analysis, we focused on the nine spatiotemporal strata that include NNCESS or SNCESS. We then condensed those nine into three strata: two defined to contain only one of the stocks (“NNCESS stratum” or “SNCESS stratum”) regardless of month and one in which both stocks can occur separately or simultaneously depending on the 2-month period (“Mixed stocks stratum”). Each sighting of each individual (from both the 2018 SNCESS and the long-term NOAA Beaufort Catalogs) was assigned to one of the three strata and compared to the sighting location of the individual during the 2018 survey by trackline area (SNCESS winter boundary, NRI_Trackline, or SC_Trackline) and by habitat (estuary or coast). For example, an individual sighted during 2018 within the SNCESS winter boundary with sightings histories in a unique NNCESS stratum would be scored as occurring in both strata.

Also, as a result of the distribution of cross-strata sightings of individuals from the 2018 survey, we specifically reviewed sighting patterns, as above, of individuals identified during a fall-winter biopsy survey we conducted in estuarine waters within the SNCESS winter boundary for five days during each November and December 2014.

### Social network analysis

As stock membership has depended on *a priori* spatiotemporal definitions, we conducted an analysis that depends only on social association patterns to identify social networks and potential population structure [60, 61]. These analyses included all of the individuals in the 2018 SNCESS and long-term NOAA Beaufort Catalogs, and were conducted with the commonly used, open-access software SOCPROG (Version 2.8) [62]. Two metrics in SOCPROG reinforce whether the data support the existence of social networks: cophenetic correlation coefficient (CCC) and modularity. CCC is the correlation between the association indices from the association matrix and the position of dyads (pairs of individuals) in a cluster dendrogram [61]. It helps with eliminating spurious clustering in a dendrogram. CCC ranges from 0 to 1, where 1 is perfect correlation between dyadic entries in the matrix of association indices and the levels at which dyads are joined in a dendrogram. The dendrogram is considered to reasonably represent the association matrix if CCC is >0.8 [61]. Modularity tests whether association indices are generally high among individuals within clusters and generally low among individuals between clusters using the difference between the estimated (observed) and expected (random) indices, thus assessing whether the social affiliations support a homogeneous or delineated community, defined as a “largely behaviorally closed set of animals in which most individuals interact” with each other [61, 63, 64]. A modularity coefficient (Q) of 0 indicates random structure; Q >0.3 is considered to result in structure that is representative of the true social structure [63]. We conducted association analyses ranging from ≥3 to ≥7 sightings per individual to find the number of sightings per individual needed to achieve at least the minimum values for CCC and modularity.

Once the number of sightings required per individual was determined, we conducted standard analyses of association using SOCPROG. We defined associations as dolphins seen in the same group during a sampling period of one day [61, 65]. Associations were tested using the half-weight index (HWI) [66], which indicates the proportion of time dyads are associated while accounting for when individuals in pairs are sighted separately. HWI ranges from 0 (never associated) to 1 (always associated). For each individual, the mean association index is the mean HWI across all its dyads and the maximum association index is the highest HWI for that individual. The sum of HWI for an individual across all associations is a measure of association strength, *i*.*e*., how well an individual is connected with other individuals; the higher the sum the greater the strength of an individual’s relationships [61, 62, 67]. Variability in the probability of association of a dyad, referred to as social differentiation (S), was measured as the coefficient of variation (CV) of HWI; S<0.3 indicates homogeneity, greater than about 0.5 indicates a well differentiated community [61]. The accuracy of the resulting social structure was tested using Pearson’s correlation coefficient (r), scaled from of 0 to 1, where 0 indicates that the analysis did not detect the true social structure. We tested the null hypothesis of no preferred associates, which is rejected if S2*H (mean number of observed associates per individual) is >5 [61]. On the basis of those results, we ran a hierarchical cluster analysis using Ward’s average linkage method [62] on the HWI association matrix, which produced a dendrogram with the clusters. A social network diagram was generated using Gephi 0.9.2. Spatial plots of the distribution of members of each cluster were plotted by season using ArcGIS. Cluster membership was compared to the designated stock membership, the latter determined by the spatiotemporal stratum in which the sighting occurred.

SOCPROG tests were run to determine if dyads were seen more or less often than expected (preferred or avoided associations) [65, 68, 69]. Preferred dyads were also assessed using the dyadic HWI [70]. Dyadic associations were tested between and within sampling periods, which indicates long-term or short-term, respectively, preferred or avoided dyads. Preferred dyads had significantly high values, interpreted as >2x the mean HWI, of the HWI relative to an expected value obtained from permutated data, while avoided dyads had significantly low values [61]. A two-tailed Mantel test was used to test whether association rates were the same within and between clusters and tested (generalized linear model, SAS Proc GLM) using the mean HWI by individual from the HWI matrix.

Standardized lagged association rates (SLAR) were run in SOCPROG to estimate temporal stability in dyads, that is, the probability that a dyad associated at one time remains associated after a time lag [61, 71]. Four exponential models were fit to the output data; the models include combinations of the duration of associations (long term (“constant companions”, CC), short term (“casual acquaintances”, CA), and disassociation [61, 71], with the best-fit model determined using the lowest quasi-Akaike Information Criterion (QAIC) [72]. Precision was estimated using jackknife resampling, wherein one observation is omitted during repeated resampling [61, 73, 74].

### *Xenobalanus* infestation and cluster membership

We examined the prevalence of *Xenobalanus* in estuarine-resident dolphin groups sighted during the 2018 survey, comparing groups sighted on the coast with those sighted in the estuary (Chi-squared, SAS Proc FREQ). In addition, after the social network analysis identified the individuals in the clusters, we counted *Xenobalanus* on the dorsal fin for all sightings of each of those individuals, excluding same-day sightings, using the photo-ID images in both the 2018 SNCESS and NOAA Beaufort Catalogs. We compared infestation levels among clusters and among individuals within clusters to examine whether *Xenobalanus* infestation was an indicator of cluster membership (S4 File).

### Abundance estimation

Following the methods of Gorgone et al. [50], we used closed (CMR) models to estimate abundance, using both (a) LTM and (b) LTM plus STM individuals. The assumption of closure is supported by the short duration of sampling, during which immigration and emigration are expected to be negligible [41] and survival of non-calves is expected to be high [75]. For analysis, each individual’s sighting history was pooled within each sampling period.

We considered four CMR model configurations that differ in how they treat sighting probability [76]. Model M_0_ is the null model for which sighting probability is assumed constant over time and across individual dolphins. Model M_t_ allows the sighting probability to differ among sampling periods (time), as might result from differences in weather or water conditions. Model M_h_ allows for each individual to have its own sighting probability (capture heterogeneity), treated as a random effect. Model M_th_ allows for time and individual effects, with time treated as additive fixed effects and capture heterogeneity treated as random effects. Although we included models with heterogeneous sighting probabilities (M_h_ and M_th_) for completeness, we note that with only three sampling occasions, such models can produce unreliable estimates [29]. For each of the four configurations, we used data augmentation to estimate abundance from the observed sighting histories [76, 77]. Data augmentation attempts to account for individuals that were present in the system but never observed [54]. To compare performance of models, we report the delta deviance information criterion (ΔDIC). The ΔDIC is computed as DIC of each model less the DIC of the best performing model, such that models with smaller ΔDIC should be preferred.

Each of the four model configurations were applied to each of four data sets, which comprised two area definitions (defined SNCESS boundary or entire survey area) and two photo-ID definitions (LTM or LTM + STM). Thus, the application design had 16 different model by data-set combinations. For data sets containing only the LTM individuals, we applied the method of Eguchi [54] to account for individuals that were present but could not be identified. In short, that method uses repeated sampling of individuals in groups to estimate the proportion (θ) of identifiable animals. It then adjusts the abundance estimate of individuals that can be identified (N_marked_) to obtain an estimate of total abundance (N_total_): ${\hat{N}}_{total}=\frac{{\hat{N}}_{marked}}{\hat{\theta }}$. Both analyses (*i*.*e*., estimation of N_marked_ and of θ) were conducted simultaneously in a Bayesian framework. For the data sets containing LTM + STM individuals, no adjustment was necessary (i.e., θ = 1) as using STM allowed for identification of all individuals in this data set.

We applied uniform prior distributions on the majority of parameters. For these, we examined posterior distributions to ensure that the lower and upper bounds of the priors were not restrictive of the estimates. The one exception to a uniform prior distribution was for the parameter controlling precision of random effects of individual sighting probabilities (Models M_h_ and M_th_). For this parameter, we applied a gamma distribution [GAM(5,1)], which improved convergence while allowing for a broad range of values, with 95% of the distribution providing variance between 0.1 and 0.6 [50].

To numerically evaluate posterior distributions of estimated quantities, Markov chain Monte Carlo (MCMC) was implemented using JAGS version 4.3.0 [78], run in R version 3.6 [79] with the R package R2jags [80]. We ran five independent Markov chains, each for 250,000 iterations. Posterior distributions were computed after a burn-in period of 100,000 iterations. We assessed convergence through visual inspection of trace, density, and autocorrelation plots, and by examining the Brooks-Gelman-Rubin statistic for values near one [81]. JAGS code used for estimation is provided in Gorgone et al. [50].

## Results

A summary of the samples used in each of the analyses below is provided in Table 1.

**Table 1:** Data sets included in the various analyses.

Analysis	Data set
Sighting histories for individuals identified during the 2018 survey	2018 photo-ID results (2018 SNCESS Catalog) compared to the long-term photo-ID catalog (NOAA Beaufort Catalog), which includes on- and off-effort sightings
Sighting histories for individuals identified during a 2014 winter survey in SNCESS winter habitat	2014 photo-ID results compared to the NOAA Beaufort Catalog
Social network analysis (SNA)	All individuals in the 2018 SNCESS and NOAA Beaufort Catalogs, including on- and off-effort
Analyses based on clusters	Individuals from the SNA with ≥7 sightings (n=95)
*Xenobalanus* load on groups	Groups sighted on-effort during the 2018 survey
*Xenobalanus* counts among individuals and clusters	All sightings in the 2018 SNCESS and NOAA Beaufort Catalogs of each of the 95 individuals included in the social network clusters
Anomalous sea-surface temperatures before and during the 2018 survey	SST collected during the 2018 survey, and SST and SST anomaly data from NOAA
Abundance estimate	On-effort sightings during the 2018 survey

### Survey effort and sightings

The three sampling sessions occurred 8–11 January, 15–19 January, and 22–26 January 2018. Survey conditions were good and all tracklines were covered as planned, except for one day of deteriorating weather that resulted in partial completion of the SC_Trackline. Sea states ranged from 0–3 except for 6.8 km over three different days at sea state 4 in coastal waters. On one day, fog limited visibility along the trackline for about 10 km in estuarine waters; nevertheless, an encounter occurred during this time. For both boats and all three sessions, 1,397 km of trackline were surveyed (785 km estuarine and 613 km coastal) within the defined SNCESS area, 12.8 km within the NRI_Trackline, and 131 km within the SC_Trackline. An additional 249 km was covered while photographing on-effort sightings. Off-effort exploratory coverage occurred for 17.7 km in estuarine waters in the lower New River estuary on one day and for 9.5 km in estuarine waters south of the Little River Inlet on one day.

There was a total of 88 on-effort sightings of presumed estuarine-resident bottlenose dolphins, 78 of which occurred within the defined SNCESS winter boundary (Table 2 and Fig 2). An additional four groups of presumed coastal migratory and seven estuarine groups sighted off-effort were excluded from all analyses specific to the 2018 survey; however, fin images meeting photo quality and distinctiveness criteria were added to the NOAA Beaufort Catalog and included in determining resighting patterns.

**Table 2:** Number of groups and individuals of estuarine-resident common bottlenose dolphins (*Tursiops truncatus*) sighted during the 2018 capture-mark-recapture abundance survey in southern North Carolina.

Parameter	Defined SNCES Boundary	NRI_Trackline	SC_Trackline	Entire survey area
No. of sightings	78	5	5	88
No. of dolphins seen	669	85	24	778
No. of unique dolphins	483	85	24	547
LTM	337	66	16	387
STM	146	19	8	168

Data for 88 on-effort sightings of common bottlenose dolphin (*Tursiops truncatus*) groups presumed to be from the Southern North Carolina Estuarine System Stock (SNCESS) during capture-mark-recapture surveys in January 2018. Sightings data are reported by location of sighting: within the SNCESS defined winter boundary, along the NRI_trackline, or along the SC_trackline. The number of dolphins represents the pool of dolphins across all sightings before photo-identification matching was completed (*i*.*e*., some animals were seen more than once during the survey). Once matching was completed, the numbers of unique dolphins were grouped by whether the match was based on short-term (STM) or long-term marks (LTM). The two groups are not additive among areas due to resightings of individuals in different areas, nor among LTM and STM, as photographs of dolphins resighted within the survey may have different photo qualities.

There were 32,261 photographs taken on-effort for the entire survey area, from which 547 unique individuals were identified (778 including resightings) across all qualities and distinctiveness ratings. Of the 547 individuals, 483 were seen only within the defined SNCESS boundary and not on the NRI_Trackline or SC_Trackline (Table 2).

**Fig 3:**
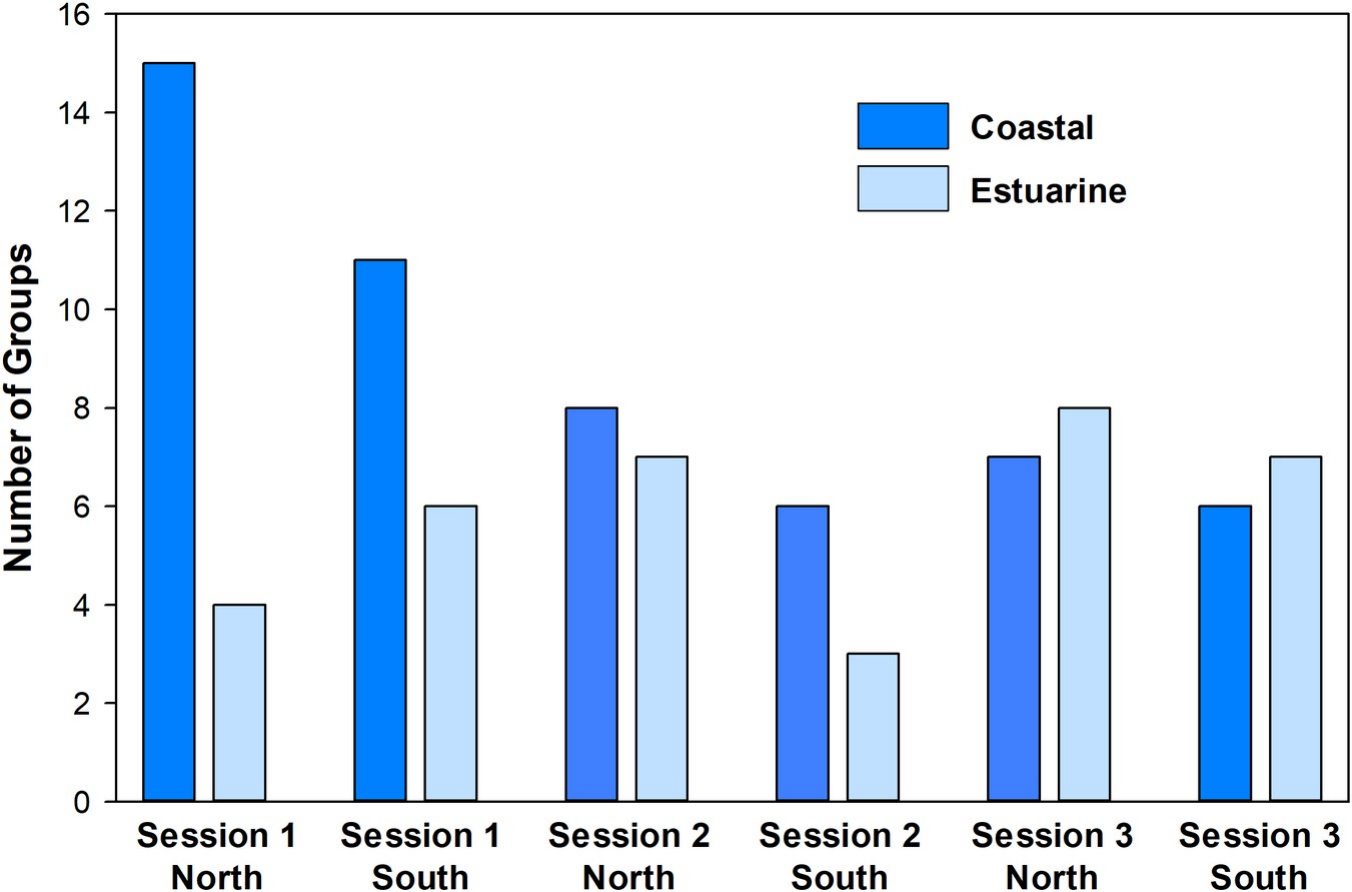
Encounters of dolphin groups during each of three survey sessions by habitat and area. More dolphin groups were encountered during the first of the three photo-ID sessions and during all sessions more sightings occurred north of Cape Fear (North) relative to south of Cape Fear (South). S1, S2 and S3 indicate the session.

The absolute number and the number of groups and individuals per km surveyed (effort-corrected) was greater in Session 1 and north of Cape Fear (Fig 3 and S1 Table). Within the defined winter boundary of SNCESS, more sightings occurred in coastal (n = 53) than estuarine (n = 35) waters, although 79% (n = 42) of the coastal groups included at least one dolphin matched to estuarine strata (from the 2018 SNCESS or NOAA Beaufort Catalog). Mean salinity was higher for encounters in coastal than estuarine waters (t-test, p<0.001) while the mean SST was slightly higher in the estuary (t-test, p = 0.048) (Table 3). No difference was detected in the mean group size (p = 0.369) or mean depth (p = 0.447) between groups sighted in the estuary and along the coast.

**Table 3:** Group and environmental characteristics during the winter 2018 abundance survey for estuarine-resident common bottlenose dolphins in North Carolina.

Characteristic	Parameter	Defined winter boundary		Extended survey area	
		Coastal	Estuary	Coastal	Estuary
Group Size	Mean	9.6	8.6	14.7	n/a
	SE	1.6	1.4	2.4	n/a
	Min	1	1	2	n/a
	Max	50	47	50	n/a
Depth (m)	Mean	5.3	4.9	5.7	n/a
	SE	0.4	0.5	0.2	n/a
	Min	1.2	1.1	2.4	n/a
	Max	15.2	15.2	7.9	n/a
Salinity (ppt)	Mean	33.2	27.8	33.8	n/a
	SE	0.3	1.3	0.1	n/a
	Min	25.6	1.1	32.4	n/a
	Max	35.1	34.9	35	n/a
SST (°C)	Mean	7.1	7.5	6.3	n/a
	SE	0.1	0.2	0.2	n/a
	Min	4.6	4.4	4.1	n/a
	Max	8.8	10.7	7.7	n/a

Characteristics of on-effort sightings of groups of estuarine-resident bottlenose dolphins during the 2018 capture-mark-recapture abundance survey. Sightings within the defined winter boundary of the Southern North Carolina Estuarine System Stock (SNCESS) are stratified by habitat of sighting (coastal, n = 43 groups; estuarine, n = 35 groups). Surveys in the extended survey area (New River Inlet trackline plus South Carolina trackline) were coastal only (n = 10 groups). Sightings of dolphins presumed to be members of the Southern Coastal Migratory Stock were excluded. n/a = not applicable. ppt = parts per thousand. SST = Sea surface temperature. SE = standard error, Min = minimum value, Max = maximum value.

### Sighting patterns relative to designated stocks

Of the 547 individuals identified during the 2018 survey, 228 matched to the NOAA Beaufort Catalog (Table 4). The 228 included individuals with STM, some persisting longer than previously assumed as indicated by matches to the long-term catalog; 34 dolphins would have been excluded from the analyses if using only LTM. Sighting histories of matched individuals ranged from 1–19 sightings over 2–23 years. Of the 228 individuals, 201 were seen within the defined SNCESS winter boundary during the 2018 survey and 93 of those had sighting histories ≥10 years. Of the 201 individuals, 40 (25%) had previously been seen only in defined spatiotemporal SNCESS strata, 160 (75%) had sightings histories in both SNCESS and NNCESS strata (S2 Fig), and one was sighted in both strata as well as in SC. During the 2018 survey, 53% of the 40 SNCESS-strata-only individuals were sighted only in the estuary, 23% sighted only on the coast, and 25% seen in both habitats, while most of the 160 individuals seen in both stock strata were sighted in coastal waters (59%), with 31% seen only in the estuary, and 11% seen in both habitats. Notably, 65 of the 160 (41%) dolphins were included in the abundance estimate for the NNCESS in summer 2013 [50], representing 14% of the individuals (total n = 471) included in that estimate. The remaining 27 (of 228) individuals were seen only in the extended survey areas; individuals sighted along the NRI_Trackline had sighting histories only in NNCESS strata, while individuals sighted along the SC_Trackline during the 2018 survey were seen previously as far north as NNCESS strata.

**Table 4:** Stock-strata assignments for estuarine-resident common bottlenose dolphins (*Tursiops truncatus*) from sighting histories.

Sighting Location 2018 Survey	SNCESS Strata	NNCESS Strata	SNCESS+ NNCESS Strata	SNCESS+ NNCESS Strata+SC	SNCESS Strata SC	Mixed Strata+SC	Overall
SNCESS	40		159	1	1		201
Coast	9		93	1			103
Estuary	21		49				70
Coast+Estuary	10		17		1		28
NRI_TrackLine		21					21
Coast		21					21
Estuary							
Coast+Estuary							
SC_Trackline				1	3	2	6
Coast				1	3	2	6
Estuary							
Coast+Estuary							
Overall	40	21	159	2	4	2	228

Location of sightings for the 228 unique common bottlenose dolphins photographed during the winter 2018 abundance survey that matched to the NOAA Beaufort long-term photo-identification catalog. Each animal was associated with two sighting locations. The first was the sighting location during the 2018 survey and the second is from the sighting history of each individual using the currently defined spatiotemporal strata for NC estuarine-resident stocks. For example, of 103 individuals seen on the coast within the SNCESS boundary during the 2018 survey, 93 had sightings histories that also included NNCESS strata. The SC (South Carolina) stratum is not a defined habitat for SNCESS and so is listed as a separate stratum.

During the November-December 2014 survey, 66 unique dolphins were identified within the SNCESS winter boundary; 57 had sighting histories in the NOAA Beaufort Catalog, spanning 1–23 years. Only 14 of the 57 (25%) had sighting histories only in SNCESS strata, similar to the rate from the 2018 survey. An additional three had sighting histories only in SNCESS strata or SC.

### Social network analysis

For the social network analysis, a minimum of seven sightings per individual was required to achieve a CCC value >0.80 (Table 5). Modularity coefficients exceeded >0.3 for all sighting levels tested (≥3 - ≥7), indicating non-random associations even at relatively low levels of repeated sightings. Seven or more sightings per individual from the 2018 SNCES and NOAA Beaufort Catalogs resulted in a sample size of 95 individual dolphins with 943 sightings; the following results are from those sightings.

**Table 5:** Effect of number of sightings on social network measures for estuarine-resident common bottlenose dolphins (*Tursiops truncatus*) in North Carolina.

Number of Sightings	Number of Dolphins	Number of Records	Number of Clusters	Modularity Coefficient	CCC
≥3	473	2500	12	0.465	0.664
≥4	290	1925	12	0.437	0.695
≥5	187	1494	6	0.437	0.74
≥6	142	1250	7	0.441	0.765
≥7	95	945	6	0.452	0.815

Results of social network analyses conducted for estuarine-resident common bottlenose dolphins in waters of North Carolina, US. The analysis was run for individuals with the number of sightings ranging from ≥3 to ≥7, with a corresponding decreasing number of unique individuals (Number of Dolphins) and the total number of sightings for those individuals (Number of Records). The Number of Clusters is the optimal number of clusters resulting from the network analysis for each number of sightings. Modularity values >0.3 indicate those clusters are representative of the true social structure. CCC, Cophenetic Correlation Coefficient, values >0.8 indicate that the resulting clusters reasonably represent the association matrix.

The overall mean association index was low (HWI = 0.04, SD = 0.02) however the maximum association indices indicated the existence of strong dyadic associations (mean 0.45, SD = 0.21, range 0.10–0.95) (S3 Fig). The sum of association indices, which approximately indicates the number of associates [77], ranged from 1.1–8.64 (mean 4.8, SD 1.84). Social differentiation (S) was 0.905, indicating a well differentiated social system. The mean association per individual (H) was 36.6, resulting in S2*H = 29.95 (above the threshold of five to reject the null hypothesis of no preferred associates). The correlation coefficient (r) between true and permuted association indices was moderate (r = 0.408). For the test for preferred or avoided associations, P-values were relatively stable from 1,000–30,000 permutations, changing only from p = 0.001 to p<0.001 for all levels of permutations; hereafter we used values from 10,000 permutations. The existence of long-term non-random associations (preferred or avoided) was supported by a significantly higher SD (0.109 vs 0.089, p<0.001) and CV (0.656 vs 0.574, p<0.001) from observed than from permuted data, respectively; avoidance of associations was supported as the proportion of non-zero elements for observed data was less than for permuted data (0.221 vs 0.258, respectively).

**Fig 4:**
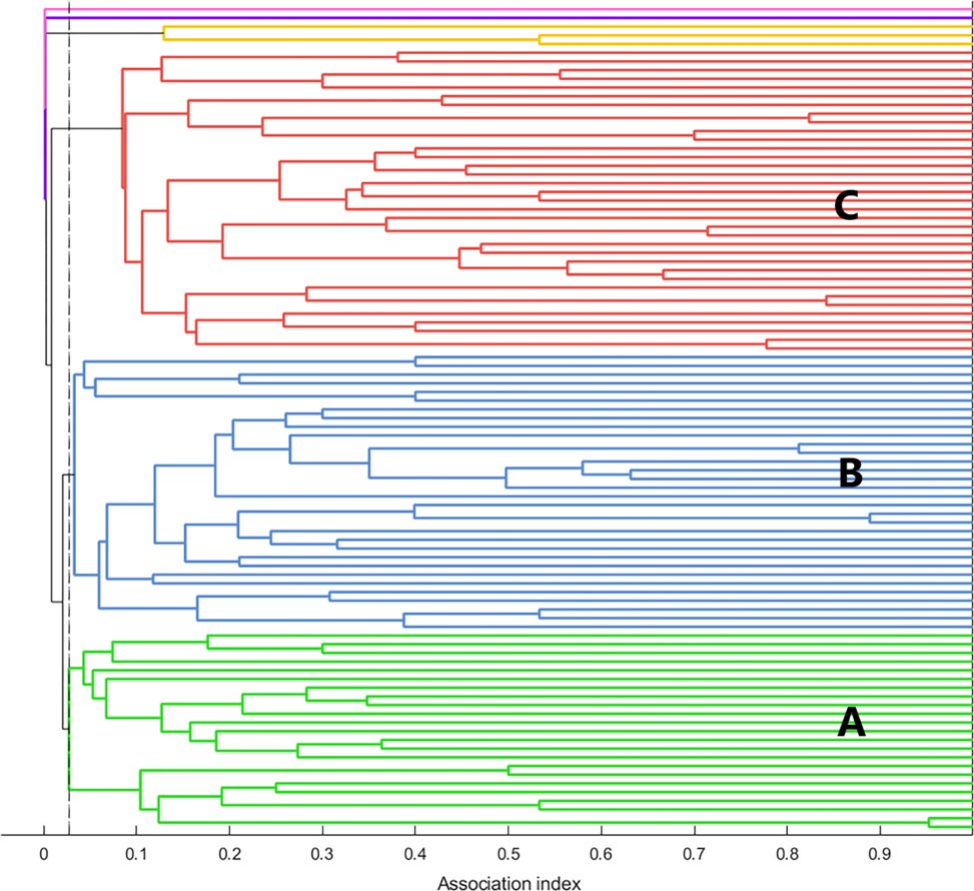
Social network dendrogram for identified clusters of estuarine-resident dolphins in North Carolina. Hierarchical cluster dendrogram from the social network analysis of estuarine-resident common bottlenose dolphins (*Tursiops truncatus*) in North Carolina). The analysis includes all dolphins with ≥7 sightings from 1995–2018, resulting in a sample size of 95 individuals and 942 sightings. Six clusters were identified. The three large clusters are labelled A-C. One cluster comprised three individuals (Cluster D, yellow lines), and two clusters comprised a single individual (Clusters E, purple, and F, pink). The dashed vertical line indicates the mean association index (HWI) and serves as the cutpoint for identifying significant clusters.

The modularity value (0.452) supported division into clusters and the CCC (0.815) indicated a good match between the dendrogram and the association indices. Six clusters (A-F) were identified (Fig 4). Clusters A-C each included 23–35 individuals while Clusters D-F included 1–3 individuals. Results from the Mantel test (t = 31.603, p<0.001) indicated that association rates within clusters (mean±sd, 0.11±0.06) were significantly greater than association rates between clusters (0.01±0.01). Association rates between pairs of clusters (with n>1) showed significantly higher within than between association rates, and included some with between-cluster association rates that were zero or low (S2 Table). The highest between-cluster summed and maximum HWI indices occurred between Clusters A and B, followed closely by Clusters B and C; the index was relatively low between Cluster A and Cluster C. For the three large clusters, Cluster C had the highest within-cluster HWI, Cluster A had the lowest.

For the three large clusters, permuted associations between clusters identified 34 significant long-term dyads (HWI≥0.08, one-sided test, p≥0.975) involving 44 individual dolphins, as some individuals had long-term associations with multiple individuals. Permutated associations within clusters identified 26 significant short-term companionships involving 32 individual dolphins. The total number of individuals in significant dyads was 58 of the 95 total individuals in the sample; 26 occurred in both long- and short-term preferred dyads. No dyads showed significant avoidance (one-sided test, p≥0.025). Most of the significant preferred dyads were in Cluster C (18 of 34 long term, 19 of 26 short-term), the fewest long-term dyads were in Cluster A (6 of 34) and the fewest short-term dyads were in Clusters A and B (3 of 26 and 4 of 26 short-term, respectively). Strength differed among the three large clusters (A-C) (generalized linear model, SAS Proc GENMOD, p<0.001 overall and for each pairwise comparison). Within-cluster strength was highest for Cluster C (mean±sd, 4.93±1.38), followed closely by Cluster B (4.05±1.61), with the lowest in Cluster A (2.43±1.10); the overall mean within-cluster strength was 3.80±1.84. Between-cluster association strength was highest between Clusters A and B (0.47±0.44), and the weakest between Clusters A and C (0.09±0.16).

**Fig 5:**
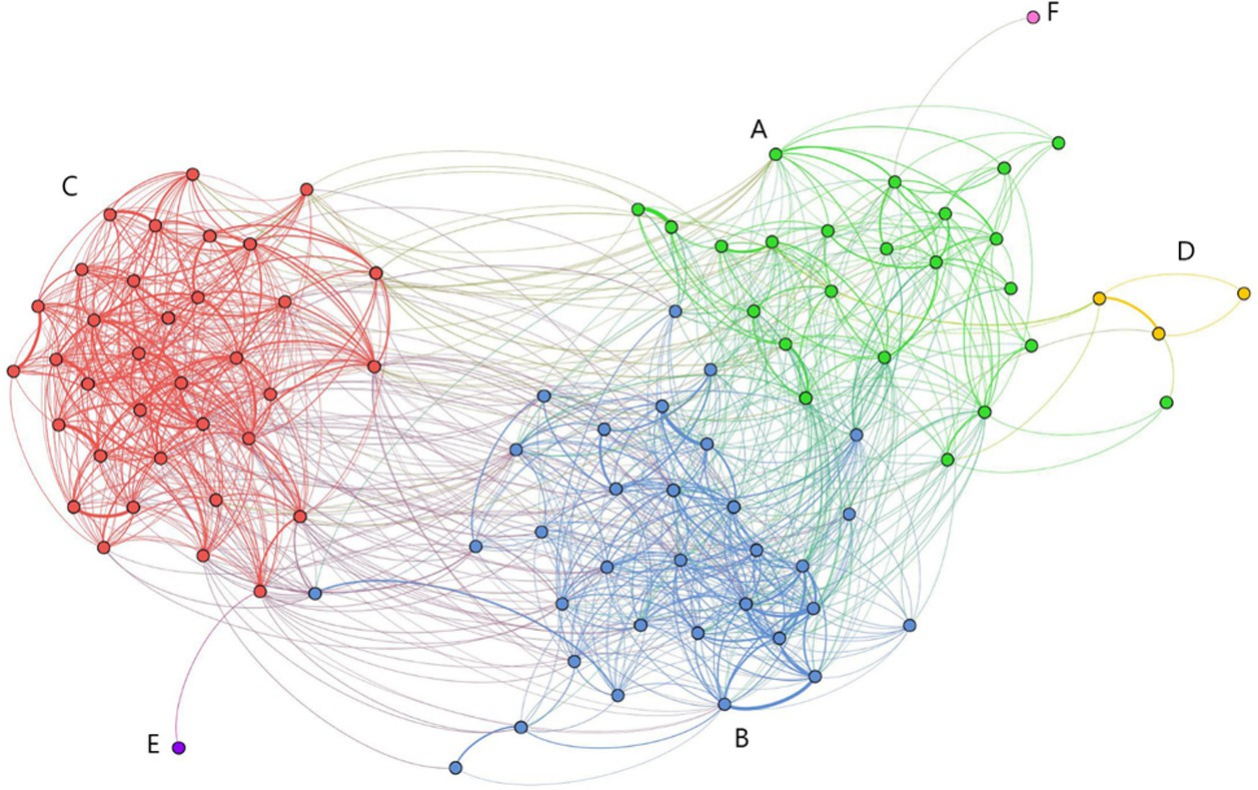
Sociogram from the social network analysis of estuarine-resident dolphins in North Carolina. Sociogram from the social network analysis of estuarine-resident common bottlenose dolphins (*Tursiops truncatus*) in North Carolina. The analysis includes all dolphins with ≥7 sightings, resulting in a sample size of 95 individuals and 942 sightings. Six clusters were identified, one of which comprised three individuals (Cluster D, yellow), and two of which comprised a single individual (Clusters E, purple, and F, pink).

The three small clusters comprised two single individuals and a group of three individuals. Sightings of the single individuals (Clusters E and F) occurred over a 10- (2006–2016) and 15-year (2003–2018) span, respectively. Cluster D included one individual sighted from 2002–2018, the second from 2003–2015, and the third only during 2002–2003. These clusters were more closely associated with Clusters A and C than Cluster B (Fig 5).

**Fig 6:**
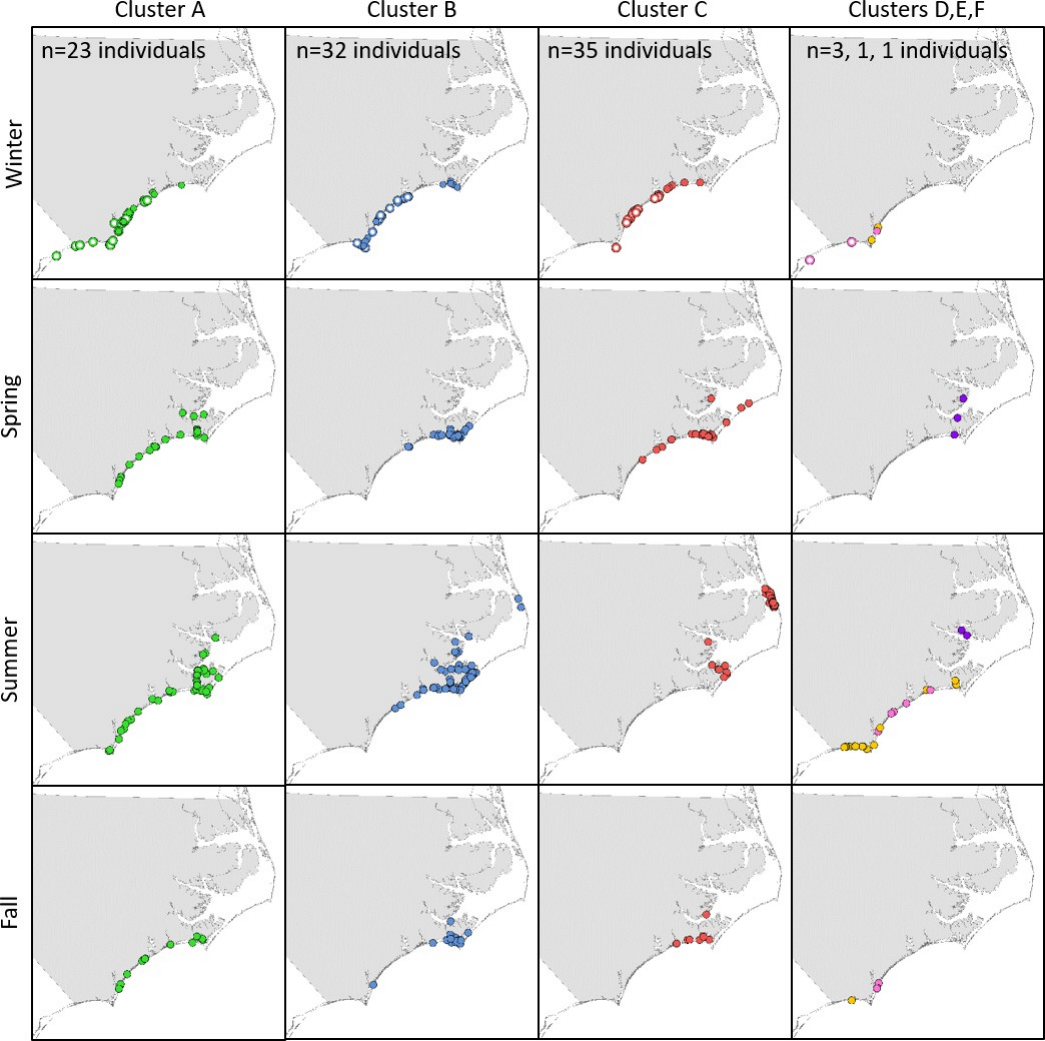
Seasonal distribution of social clusters of estuarine-resident common bottlenose dolphins. The seasonal distribution of all sightings of individual estuarine-resident common bottlenose dolphins (*Tursiops truncatus*) in North Carolina for each of the six clusters identified from the social network analysis. Sightings during the current survey are represented by open circles. Cluster D–yellow circles, Cluster E–purple circles, Cluster F–pink circles.

The proportion of each cluster that included individuals sighted during the 2018 survey varied (Table 6). Cluster A included the highest proportion (22 of 23 individuals). About half of the other two large clusters included individuals sighted during the 2018 survey (Cluster B: 17 of 32, Cluster C: 19 of 35). Of the three small clusters, two had no individuals (Cluster D: 0 of 3 and Cluster E: 0 of 1) seen during the survey while the single animal from Cluster F was seen. None of the large clusters corresponded to a currently designated stock (Fig 6); although the proportion of sightings of individuals in Clusters B and C was higher within NNCESS strata while those in Cluster A were higher in SNCESS strata.

**Table 6:** Correspondence between cluster assignment and sighting locations of individuals within each cluster.

Cluster	Number of Individuals	Number of Sightings	Proportion of Sightings		
			NNCESS	SNCESS	Mixed
A	23	237	0.25	0.52	0.23
B	32	310	0.7	0.15	0.15
C	35	355	0.74	0.14	0.12
D	3	23	0	1	0
E	1	11	1	0	0
F	1	7	0	1	0

The proportion of sightings of individuals within each cluster that occurred within the defined stock areas for NNCESS, SNCESS, or the mixed-stock stratum.

While there were seasonal shifts in distribution within clusters, co-occurrence among clusters existed within all seasons (Fig 6). Nonetheless, some differences in distribution occurred among clusters in summer and they were consistent with differences in between-cluster association rates (for cluster size >1). Individuals in Cluster C were seen frequently and those in Cluster B seen occasionally in northern Pamlico Sound, within the range of NNCESS. Two clusters (A and D) never seen in northern Pamlico Sound were also the only two clusters seen in southern NC during the summer. For the three large clusters and across all seasons, all individuals were sighted at least once in the estuary and no individuals were sighted only on the coast. However, more individuals in Clusters A (65%) and B (69%) relative to Cluster C (31%) had coastal sightings (n = 90 individuals in Clusters A–C, Chi-squared p = 0.004), with no difference between Clusters A and B (Chi-squared, p = 0.783). Similarly, across all sightings of each individual in Clusters A-C (n = 900 total sightings), 12–13% of those in Clusters A and B and 3.7% of those in Cluster C were coastal.

The basic structure of the dendogram changed little when the number of sightings required for inclusion was reduced to as low as ≥3 sightings per individual. For individuals with ≥4 to ≥7 sightings, there were three larger clusters. Although the total number of clusters increased with increased sample size (*i*.*e*., fewer required sightings for inclusion), the clustering pattern for dendograms consistently showed two primary higher-level nodes (equivalent to Cluster A nested with Cluster B and Cluster C nested separately). Thus, the reduced sample size due to using individuals sighted only ≥7 times did not substantially affect the basic cluster pattern.

The SLAR for temporal patterns of association was significantly higher than expected by chance (null model), supporting long-term dyadic associations (S4 Fig). The SLAR declined over time but stabilized above the null rate. The best performing model included “preferred companions” and “casual acquaintances”.

### *Xenobalanus* infestation

For dolphin groups sighted during the 2018 survey, *Xenobalanus* load varied by habitat of sighting, with a significantly higher infestation on coastal groups (Chi-squared, p<0.001) (S4 File). For the 90 individuals in Clusters A-C (n = 825 sightings), *Xenobalanus* was significantly more prevalent in coastal sightings and during winter and spring. Cluster was not a significant predictor overall for *Xenobalanus* prevalence (p = 0.07).

### Abundance estimation

Among the two habitat areas (estuarine and coastal) and for both LTM and LTM/STM categories, the highest number of resightings between at least two sessions was 10–11% of the overall number of unique individuals (Table 7). Between 4–5% of unique dolphins were seen across all three sessions. Each CMR model demonstrated adequate convergence of MCMC chains on each data set, including all Brooks-Gelman-Rubin statistics near 1.0. For each data set, ΔDIC indicated that Model M_t_ performed best, followed in rank by M_0_, M_th_, and M_h_. This provides support that sighting probabilities vary through time (M_t_ compared to M_0_, and M_th_ compared to M_h_), but model performance did not improve with inclusion of heterogeneity in sighting probability.

**Table 7:** Individually identified dolphins within and between sessions of a capture-mark-recapture survey of estuarine-resident bottlenose dolphins (*Tursiops truncatus*) in southern North Carolina.

	LTM		LTM & STM	
Sessions	Defined winter boundary	Entire survey area	Defined winter boundary	Entire survey area
S1	187	218	269	316
S2	102	127	141	170
S3	126	140	174	190
All Sessions	337	387	476	547
Resightings				
S1 & S2	28	36	41	49
S2 & S3	30	39	42	52
S1 & S3	36	39	46	49
S1 & S2 & S3	16	16	21	21

Number of individually identified bottlenose dolphins within each session (S1-S3) of the 2018 survey and number of dolphins resighted among sessions within the defined winter boundary for the Southern North Carolina Estuarine System Stock and across the entire survey area (New River, NC, to Murrell’s Inlet, SC). Totals are shown for all dolphins categorized as having long-term marks (LTM), and for all identified dolphins (LTM and short-term marks [STM]). Numbers of unique dolphins in the three sessions are not additive because of resightings.

Across models and data sets, estimates of abundance, as indicated by medians of posterior distributions, ranged from 806 to 1520 animals (Table 8). Models with heterogeneity in sighting probability estimated higher abundances than those without; however, we reiterate that with only three sampling occasions, those models with random effects can produce unreliable estimates [29]. Each model estimated higher abundance when using all marked individuals (LTM + STM) than when using only individuals with long-term marks (LTM) and adjusted for unidentified animals [54]. For Models M_h_ and M_th_, that pattern was reversed, with higher estimates of abundance for data sets containing all marked individuals. The best performing model, M_t_, estimated abundance (posterior medians) in the range of 806 to 1053 animals, depending on the data set.

**Table 8:** Estimates of abundance from a winter 2018 capture-mark-recapture survey of estuarine-resident bottlenose dolphins (*Tursiops truncatus*) in southern North Carolina.

Data Set (Photo ID; Area)	Model	ΔDIC	Abundance
LTM	M_t_	0	806 (684, 971)
Defined winter boundary	M_0_	194	843 (712, 1021)
	M_th_	1073	986 (773, 1606)
	M_h_	2713	1152 (834, 2452)
LTM	M_t_	0	876 (755, 1034)
Entire survey area	M_0_	200	910 (781, 1079)
	M_th_	656	1023 (839, 1406)
	M_h_	1263	1089 (879, 1639)
LTM + STM	M_t_	0	941 (827, 1086)
Defined winter boundary	M_0_	324	986 (863, 1146)
	M_th_	3091	1218 (950, 2143)
	M_h_	4914	1520 (1043, 2722)
LTM + STM	M_t_	0	1053 (937, 1199)
Entire survey area	M_0_	322	1103 (977, 1262)
	M_th_	2574	1288 (1054, 1998)
	M_h_	4518	1435 (1120, 2557)

Estimates of abundance of estuarine-resident bottlenose dolphins from data collected during capture-mark-recapture surveys in winter 2018. Four models were used. Model M_0_ is the null model for which sighting probability is assumed constant over time and across individual dolphins. Model M_t_ allows the sighting probability to differ among sampling periods (time), as might result from differences in weather or water conditions. Model M_h_ allows for each individual to have its own sighting probability (capture heterogeneity), treated as a random effect. Model M_th_ allows for time and individual effects, with time treated as additive fixed effects and capture heterogeneity treated as random effects. Photo ID refers to individuals identified using long- (LTM) or short-term (STM) marks based on photo-quality and distinctiveness criteria. Areas include the defined winter boundary for the Southern North Carolina Estuarine System Stock (SNCESS) and across the entire survey area (New River, NC, to Murrell’s Inlet, SC). Estimates shown are median values (95% credible intervals) from the posterior distributions. Also shown is the delta deviance information criterion (ΔDIC; ΔDIC = 0 for the preferred model). Note that ΔDIC is only comparable across models for the same data set (i.e., same Photo ID and Area).

## Discussion

Our results indicate that the structure of estuarine-resident bottlenose dolphins in North Carolina is more complex than previously defined. The use of a long-term photo-ID catalog provided an opportunity to compare sightings during one survey to historical sightings, use those results in a social network analysis, then compare the resulting social clusters to the currently designated management stocks. The three large clusters identified from the social network analysis appear to have been persistent. *Xenobalanus* infestations, a possible indicator of cluster membership, differed among clusters albeit they did not define them. However, the differences suggested that there were different seasonal use of habitats, estuarine or coastal, among clusters. One possible explanation for differences between cluster assignments and current stock designation, namely, anomalously cold water temperatures in 2018 (S2 File), did not appear to account for differences between the cluster assignments and current stock designation, and a 2014 winter survey supported the results from the 2018 survey. Thus, a reconsideration of stock designation for management of estuarine-resident bottlenose dolphin in North Carolina seems warranted.

Spatiotemporal strata did not define the clusters. However, the three large clusters did show general differences in primary sighting locations and will be referred to on that basis: the Pamlico Sound cluster (Cluster C, more northern and estuarine), the Beaufort cluster (Cluster B, central), and the southern NC cluster (Cluster A). These results do not negate the existence of two stocks of estuarine-resident bottlenose dolphins in North Carolina, as currently designated. Concurrence of fine-scale genetic and social structure in *T*. *truncatus*, as well as *T*. *aduncus*, has been reported in estuarine and coastal waters [e.g., 5, 36, 82–92]. In contrast, social structuring has been reported in the absence of detected genetic differentiation [9, 25, 93, 94]. There are three possible interpretations of the current results. First, per the current stock designation, the three clusters may comprise two stocks, with the Pamlico Sound cluster comprising one stock and the Beaufort and Southern NC clusters another, based on between-cluster association indices, similar use of coastal waters, and amount of spatial overlap, or the Southern NC cluster comprising one stock and the other two comprising one stock as the proportion of sightings of individuals in Clusters B and C was higher within NNCESS strata while those in Cluster A were higher in SNCESS strata. Second, it is possible that the three clusters identified form three DIPs and could, therefore, be designated as three stocks. Third, it is possible that the clusters are not DIPs and represent one stock given that the spatiotemporal overlap of the three large clusters occurs during the peak breeding seasons in spring and fall (Fig 6) [43, 95, 96] and given the level of associations documented among clusters (Fig 5). The most appropriate stock designation will require additional data, such as genetic analysis and more expansive year-round sampling, and considering that multiple DIPs can be managed as a single stock.

Spatial segregation often occurs in socially or genetically structured groups of bottlenose dolphins [91]. Spatial segregation in common and Indo-Pacific bottlenose dolphins has been identified within estuarine systems, embayments, or lagoons [e.g., 7, 11, 12, 82, 87, 91, 93, 97–100], between or among estuarine and coastal groups [e.g., 8, 14, 84, 99–104], as well as in coastal open-water areas [9, 94, 104]. The spatial segregation often reflects site fidelity and occurs despite the lack of physical barriers to movements [e.g., 7, 88, 94, 100, 105]. In contrast, some studies have found substantial overlap in core areas [15, 106] or no spatial segregation among socially [10, 93] or genetically differentiated [10, 107] groups. Griffin et al. [36] found no genetic differentiation among samples in three connected estuarine areas on the basis of sampling location only, but found genetic division when samples from bottlenose dolphins with ≥ 10 sightings were assigned to the region where ≥ 50% of those sightings occurred. Cantor et al. [107] found that spatial distribution did not influence the probability of individuals associating, with kernel density showing three clusters in a nested pattern in an area along the east coast of Brazil. Chabanne et al. [8] also found overlapping home ranges that did not explain association patterns in Indo-Pacific bottlenose dolphins in western Australia. The distribution of clusters of estuarine-resident dolphins in NC are more similar to the latter situations, with substantially overlapping ranges and spatial distribution not driving associations. It has been proposed that the maintenance of associations or social groups is a function of cultural transmission [e.g., 2, 85, 99, 108–113], and that may be the most parsimonious explanation for social structuring in estuarine-resident dolphins in NC.

Within the context of site fidelity and spatial segregation, bottlenose dolphins do intermittently move between estuarine and coastal habitats [29, 36, 114–120], with few reported exceptions [84, 98]. Wells et al. [14] and Takeshita et al. [121] found three typical movement patterns for estuarine-resident bottlenose dolphins satellite tagged in Barataria Bay, Louisiana (Gulf of Mexico, US), and its barrier islands; two to three genetic clusters were also identified [89]. The movement patterns were associated with different residency patterns and habitat features [14, 121], differing by the percentage of locations solely from estuarine or from coastal waters, and for the latter, and average distance from shore of movements. The highest use of coastal waters, 36% of locations, was by dolphins found primarily around the barrier islands and inlets. The distribution patterns and use of more coastal habitat by individuals from the southern NC and Beaufort clusters suggest use of coastal habitats more similar to the barrier island group described by Wells et al. [14]. Individuals in the southern NC and Beaufort clusters, however, were primarily sighted where the estuarine system is a narrow corridor relative to both the open waters of Barataria Bay and the expansive estuarine habitat in central and northern NC where dolphins seem to use estuarine waters primarily. This feature of being an estuarine corridor with many inlets may provide a relatively unique habitat for bottlenose dolphins, resulting in greater than expected use of coastal waters by estuarine-resident dolphins. One result is that members of the two clusters may occur coastally more often than expected for estuarine-resident dolphins, although the telemetry results from Wells et al. [14] may require more expansive thinking about the complexity of habitat-use by bottlenose dolphins.

The strength of associations both within and between dolphin groups is influenced by a number of factors [122]. Within groups, and within the context of the fission-fusion societies typical of bottlenose dolphins [1], more cohesion has been found in estuaries or embayments, particularly for those groups with high site fidelity and year-round residency [8, 31]. Possibly counterintuitively, relatedness, alone, has been shown to not explain the strength of social groups [8, 10, 86]. Between groups, geographic distance and overlap in home ranges have positively correlated with association strength [88, 123], but not consistently [8, 124]. Results from the current study also show higher within-cluster association strength for the Pamlico Sound cluster, most associated with the large estuarine system of Pamlico Sound, although within-cluster association strength was also high in the Beaufort cluster. In contrast, within-cluster association strength in the Southern SC cluster was low. This southernmost, coastally oriented cluster was most associated with the currently designated SNCESS. Low within-cluster association strength, along with sightings of putative SNCESS individuals along the South Carolina coast [current study, 52] suggest the possibility that sampling of the Southern NC cluster primarily in NC is not capturing the entire social unit.

In the current study, the occurrence of three small clusters (D, E, and F) with individuals sighted over long periods of time suggests a residency pattern not directly associated with the three large clusters. These individuals may be members of nearby stocks or “occasional residents” [125]. Transients or seasonal residents are a common feature for coastal and estuarine bottlenose dolphins [32, 36, 118, 126–131]. However, all of the individuals in the three small clusters ([7–11] sightings from 2002–2018) were seen in at least two seasons, with two (in Clusters E and F) seen in all four seasons. Most of the sightings of the four individuals in Clusters D and F were in southern NC, with one sighting in SC. As individuals from the designated SNCESS have been identified in the northern range of the next designated stock to the south, the Northern South Carolina Estuarine System Stock (NSCESS) [52], individuals in those two clusters may be members of the NSCESS stock, suggesting significant and frequent coastal ranging of these estuarine stock(s). Sightings of the single individual in Cluster E would all have been assigned to NNCESS strata so the reason for the formation of a new cluster is unknown.

### *Xenobalanus* infestation

Differences in *Xenobalanus* load and counts among clusters support the cluster results. It also supports the influence of salinity on *Xenobalanus* prevalence (S4 File). Members of the Pamlico Sound cluster were seen with *Xenobalanus* only during winter and spring when they were on or near the coast. Members of the Southern NC cluster were also infested during summer, supporting more year-round coastal use by this cluster.

### Abundance estimation

The original impetus for the current survey was to obtain an abundance estimate for the currently designated SNCESS stock during a period (winter) of presumed limited overlap with other stocks. However, the occurrence of dolphins identified as part of the NNCESS demonstrated that the abundance estimate was not of a single currently designated stock and not easily correctable as (a) the sightings of NNCESS individuals does not seem due to a spurious distribution resulting from anomalous water temperatures, and (b) the sightings are consistent with NNCESS sightings during our prior 2014 fall-winter biopsy survey in the defined winter habitat of SNCESS, suggesting that the 2018 results were not anomalous. In addition, more than half of the individuals included in the abundance estimate from the 2018 survey with sighting histories in NNCESS strata were also included in the most recent NNCESS abundance estimate [50]. The two abundance estimates were similar. Thus, interpretation of the current estimate, and the prior estimate for NNCESS, is not straightforward, and the current estimate cannot be construed as an abundance estimate of the currently designated SNCESS.

Silva et al. [52] estimated abundance for the presumed SNCESS from summer and winter surveys in 2014, although the surveys could not be completed as planned and the results are not usable for management [27]. Nonetheless, their winter estimate of 206 (CI 100–423) was much less than the estimate from the current study. Two prior estimates of abundance for SNCESS were conducted in summer [40, 132], when a portion of SNCESS is expected to have moved to more northerly estuarine areas, overlapping with NNCESS. In theory, the winter surveys would generate a higher abundance estimate when stocks are non-overlapping. These various abundance estimates are not comparable for other reasons, as well, including area covered and substantial differences among survey design and implementation. The difference also cannot be explained by use of LTM+STM as the estimate using both, which resulted in all individuals being identified, is still much less than the difference between current and prior estimates. It is not likely that the increase in abundance between the current survey conducted in 2018 and the 2014 winter survey [52] is due to growth in the local dolphin population in the intervening four years given a maximum growth rate of 4% [133].

The lack of a distinct spatiotemporal distribution pattern of the clusters or currently designated stocks poses challenges in obtaining abundance estimates for NC estuarine-resident dolphins. This challenge is not unique to North Carolina. Louis et al. [9] identified three social clusters of common bottlenose dolphins along the Normandy coast not completely isolated from each other and so estimated abundance for all three clusters combined rather than individually. Chabanne et al. [134] applied a multistate capture–recapture robust design to obtain abundance estimates for a metapopulation of Indo-Pacific bottlenose dolphins in western Australia, obtaining an abundance estimate for each of the three communities. Their approach of multi-season surveys across the geographic range of the communities and accounting for probabilities of dolphins moving between estuarine and coastal habitats may be needed for estuarine-resident dolphins in NC. However, this approach may be subject to scale. Chabanne et al. [134] surveyed an area of 275 km^2^. In contrast, the Pamlico Sound estuary alone is 5,335 km^2^ [47], requiring about 4,800 km of trackline excluding coastal waters [50]. The current survey covered over 1,500 km of trackline. In addition, there is habitat between those two surveyed areas that would need to be included. Thus, the Chabanne et al. [134] approach would require a substantial increase in sampling effort, both spatially and temporally, in North Carolina.

## Conclusions

Among common bottlenose dolphins inhabiting sounds, bays, and estuaries or nearshore coastal waters of the US Atlantic and Gulf of Mexico coasts, it is increasingly evident that the complexity of population or social structure is challenging the determination of appropriate units needed for management. Habitat partitioning on small spatial scales has been identified or confirmed by recent genetic, telemetry, and photo-ID results [14, 135, 136], as has been mixing of genetically distinct groups within those small areas [36]. In the US, stocks have yet to be designated using results from social network results, however, given the relatively small size of many stocks or communities of bottlenose dolphins, Chabanne et al. [25] and Bonneville et al. [7] suggested that each social community, although not necessarily demographically independent, may still warrant separate management considerations due to different exposers to anthropogenic threats. Ultimately, however, a reevaluation of the stock designation of estuarine-resident common bottlenose dolphins in NC is needed. And, regardless of the final designation of stocks or management units, the degree of spatiotemporal overlap in NC, and elsewhere, will require novel approaches to determine how to best to obtain reliable estimates of abundance and to assign anthropogenic mortality to stock.

## Supporting information

S1 Table Sightings per km surveyed (effort-corrected sightings). (PDF)

S2 Table The Half-Weight Index (HWI) of association from the social network analysis. (PDF)

S1 Fig Enlarged view of the offset tracklines within the defined SNCESS winter coastal habitat. (PDF)

S2 Fig Seasonal distribution of sightings of presumed SNCESS dolphins identified during the 2018 survey. (PDF)

S3 Fig Distribution of association indices. (PDF)

S4 Fig Standardized lagged association rate. (PDF)

S1 File Abundance, Potential Biological Removal level, and bycatch of estuarine-resident bottlenose dolphins in North Carolina. (PDF)

S2 File Analysis of anomalous sea-surface temperatures as a possible driver for the unexpected distribution of estuarine-resident bottlenose dolphins. (PDF)

S3 File Criteria for images and examples of dorsal fins with long-term marks (LTM) and short-term marks (STM). (PDF)

S4 File Prevalence of *Xenobalanus globicipitis* on estuarine-resident common bottlenose dolphins in NC. (PDF)
