# Supplementary material for: Patterns of association and distribution of estuarine-resident common bottlenose dolphins (Tursiops truncatus) in North Carolina, USA
Source: PLoS One. 2022 Aug 15;17(8):e0270057. doi: 10.1371/journal.pone.0270057 (PMC9377618; doi:10.1371/journal.pone.0270057)
Supplement: S1 Table — (PDF) [file pone.0270057.s001.pdf]

S1 Table. Sightings of estuarine resident bottlenose dolphins per km surveyed (effort-corrected sightings).

S1 Table for Hohn et al. Patterns of association and distribution of estuarine-resident common bottlenose dolphins (*Tursiops truncatus*) in North Carolina, USA

The number of dolphin groups and individual dolphins seen per photo-sampling session on the coast and in the estuary corrected for km of trackline surveyed on-effort. North and South are relative to Cape Fear. n=53 coastal groups, 35 estuarine groups, and 547 individual dolphins.

Session and Area	Coast Groups	Coast Dolphins	Estuary Groups	Estuary Dolphins	Both habitats combined: Groups	Both habitats combined: Dolphins
Session 1						
North	0.16	1.78	0.03	0.47	0.08	0.97
South	0.16	1.74	0.06	0.69	0.1	1.12
Session 2						
North	0.08	1.16	0.04	0.35	0.06	0.65
South	0.05	0.18	0.03	0.16	0.04	0.17
Session 3						
North	0.08	1.13	0.05	0.34	0.06	0.63
South	0.05	0.2	0.08	0.35	0.06	0.26
Overall	0.09	0.92	0.05	0.39	0.06	0.62
