# Supplementary material for: Patterns of association and distribution of estuarine-resident common bottlenose dolphins (Tursiops truncatus) in North Carolina, USA
Source: PLoS One. 2022 Aug 15;17(8):e0270057. doi: 10.1371/journal.pone.0270057 (PMC9377618; doi:10.1371/journal.pone.0270057)
Supplement: S2 Table — (PDF) [file pone.0270057.s002.pdf]

**S2 Table. The Half-Weight Index (HWI) of association from the social network analysis.**

S2 Table for Hohn et al. Patterns of association and distribution of estuarine-resident common bottlenose dolphins (*Tursiops truncatus*) in North Carolina, USA

The Half-Weight Index (HWI) of association (mean, sum and maximum) within and between pairs of clusters, and overall. Pairwise comparisons for which HWI was zero for all measures were excluded.

| Cluster(s) | Number of Individuals in Cluster | HWI Mean (SD) | HWI Sum (SD) | HWI Max (SD) |
|---|---|---|---|---|
| A | 23 | 0.03 (0.01) | 3.43 (1.10) | 0.38 (0.21) |
| B | 32 | 0.04 (0.02) | 5.05 (1.61) | 0.43 (0.21) |
| C | 35 | 0.05 (0.01) | 5.93 (1.38) | 0.55 (0.16) |
| D | 3 | 0 (0.00) | 1.1 (0.00) | 0.1 (0.00) |
| E | 1 | 0 (0.00) | 1.14 (0.00) | 0.14 (0.00) |
| F | 1 | 0.01 (0.00) | 1.76 (0.44) | 0.4 (0.23) |
| A:B | | 0.02 (0.02) | 0.47 (0.44) | 0.12 (0.07) |
| A:C | | 0 (0.01) | 0.09 (0.16) | 0.04 (0.05) |
| A:D | | 0 (0.00) | 0.1 (0.00) | 0.1 (0.00) |
| A:F | | 0.01 (0.01) | 0.19 (0.22) | 0.12 (0.11) |
| B:C | | 0.01 (0.01) | 0.41 (0.40) | 0.12 (0.05) |
| C:E | | 0 (0.00) | 0.14 (0.00) | 0.14 (0.00) |
| Within | | 0.11 (0.06) | 4.12 (1.77) | 0.46 (0.21) |
| Between | | 0.01 (0.01) | 0.68 (0.57) | 0.14 (0.07) |
| Overall | | 0.04 (0.02) | 4.8 (1.84) | 0.45 (0.21) |
