# Supplementary material for: Patterns of association and distribution of estuarine-resident common bottlenose dolphins (Tursiops truncatus) in North Carolina, USA
Source: PLoS One. 2022 Aug 15;17(8):e0270057. doi: 10.1371/journal.pone.0270057 (PMC9377618; doi:10.1371/journal.pone.0270057)

S1 Fig. Enlarged view of the tracklines within the defined SNCESS winter coastal habitat.

S1 Fig. for Hohn et al. Patterns of association and distribution of estuarine-resident common bottlenose dolphins (*Tursiops truncatus*) in North Carolina, USA.

Coastal tracklines (red lines) were offset randomly for each of the three photo-sampling sessions. The blue stratum in coastal waters extends 3km from shore, representing the established boundaries for the SNCES Stock in coastal waters during the month of the surveys. Green lines illustrate the planned tracklines in estuarine waters. A trackline parallel to shore was conducted along the part of the South Carolina coast. The unshaded area at the north end is outside the defined winter boundary of SNCESS; the red track line indicates surveys (NRI_Trackline) from the inlet to the start of the of the trackline within the winter boundary for SNCESS.

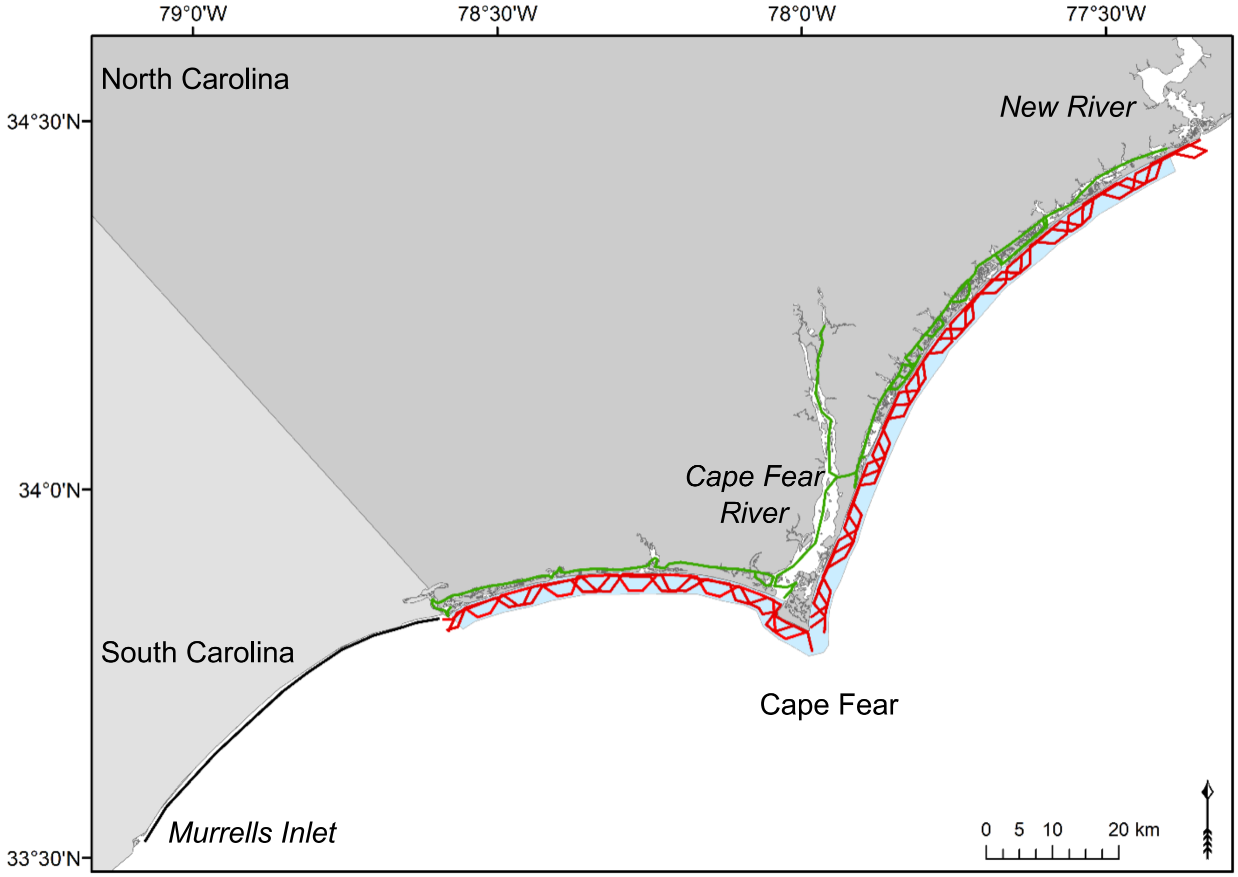

Supplement: S1 Fig — (PDF) [file pone.0270057.s003.pdf]
