# Supplementary material for: Patterns of association and distribution of estuarine-resident common bottlenose dolphins (Tursiops truncatus) in North Carolina, USA
Source: PLoS One. 2022 Aug 15;17(8):e0270057. doi: 10.1371/journal.pone.0270057 (PMC9377618; doi:10.1371/journal.pone.0270057)

S2 Fig. Seasonal distribution of sightings of presumed SNCESS dolphins identified during the 2018 survey.

S2 Fig for Hohn et al. Patterns of association and distribution of estuarine-resident common bottlenose dolphins (*Tursiops truncatus*) in North Carolina, USA.

Sighting locations for each of the 201 dolphins sighted within the defined winter habitat of SNCESS and with prior sightings in the NOAA Beaufort photo-id catalog.

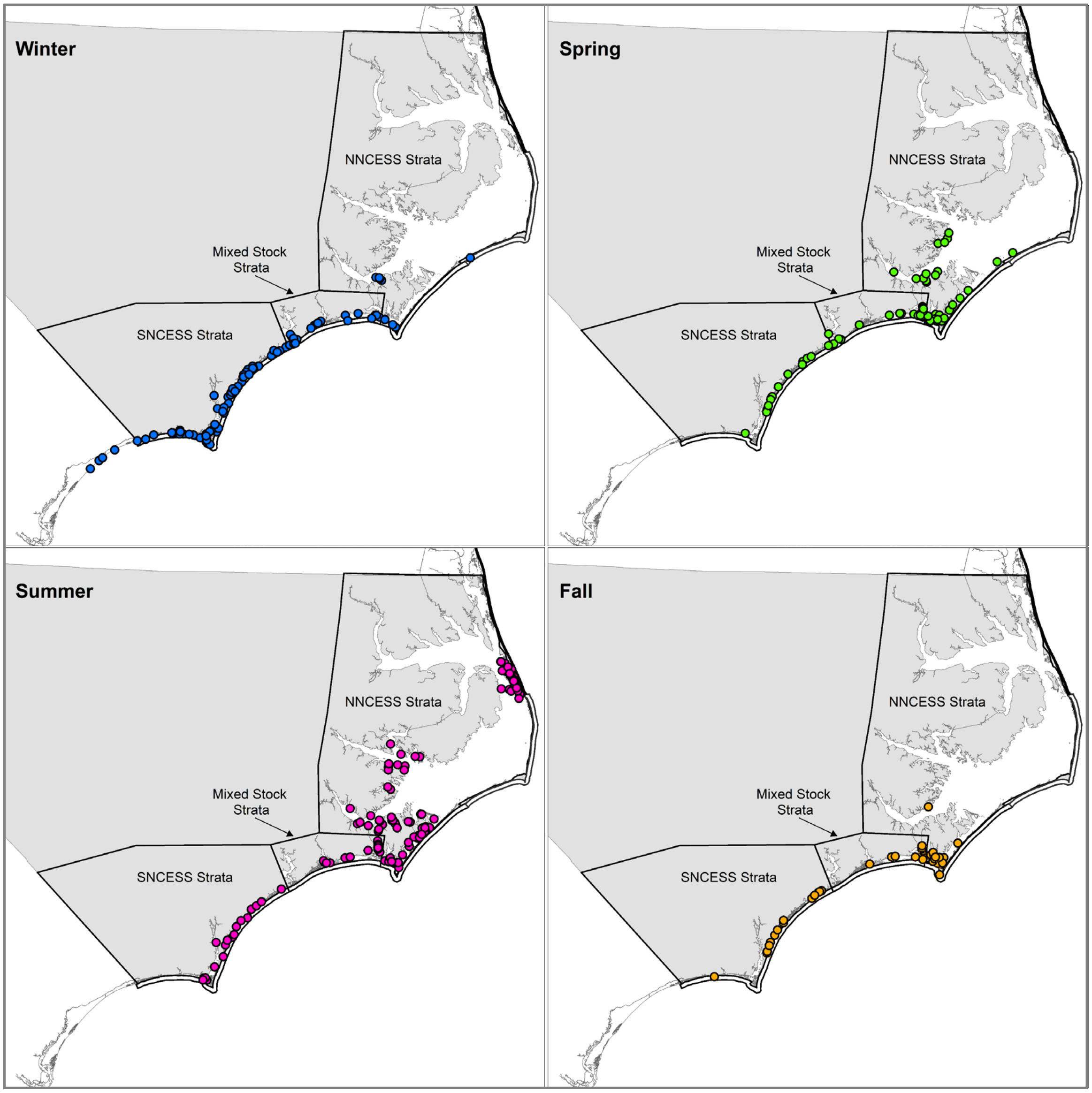

Supplement: S2 Fig — (PDF) [file pone.0270057.s004.pdf]
