# Supplementary material for: Patterns of association and distribution of estuarine-resident common bottlenose dolphins (Tursiops truncatus) in North Carolina, USA
Source: PLoS One. 2022 Aug 15;17(8):e0270057. doi: 10.1371/journal.pone.0270057 (PMC9377618; doi:10.1371/journal.pone.0270057)

S3 Fig. Distribution of association indices.

S3 Fig. for Hohn et al. Patterns of association and distribution of estuarine-resident common bottlenose dolphins (*Tursiops truncatus*) in North Carolina, USA.

Distribution of mean, sum and maximum Half Weight Index association indices for the 93 estuarine-resident bottlenose dolphins in the four clusters (A, B, C, D) with membership >1. The colors represent clusters: green=Cluster A, blue-Cluster B, Red=Cluster C, Yellow=Cluster D.

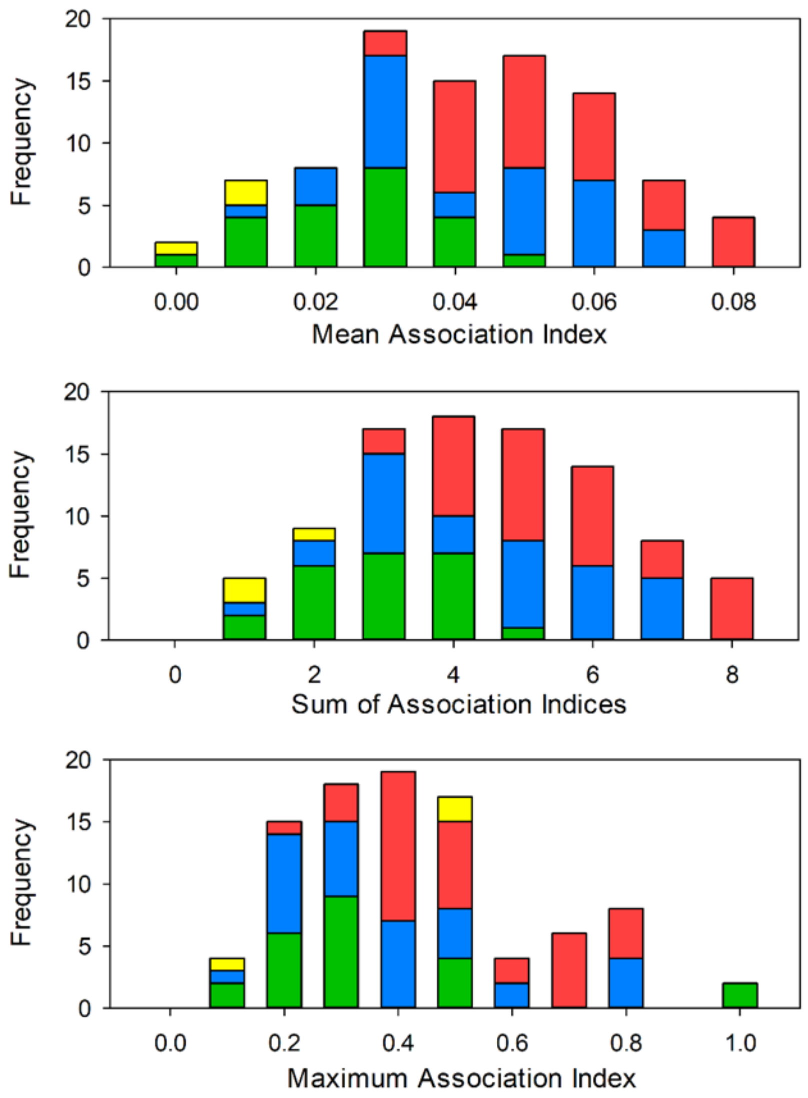

Supplement: S3 Fig — (PDF) [file pone.0270057.s005.pdf]
