# Supplementary material for: Patterns of association and distribution of estuarine-resident common bottlenose dolphins (Tursiops truncatus) in North Carolina, USA
Source: PLoS One. 2022 Aug 15;17(8):e0270057. doi: 10.1371/journal.pone.0270057 (PMC9377618; doi:10.1371/journal.pone.0270057)

S4 Fig. Standardized lagged association rate.

S4 Fig for Hohn et al. Patterns of association and distribution of estuarine-resident common bottlenose dolphins (*Tursiops truncatus*) in North Carolina, USA

Standardized lagged association rate (SLAR) for 95 estuarine-resident common bottlenose dolphins in North Carolina, showing the observed and model-fit. Standard errors on observed rates are shown as vertical bars. The best fit model indicates a social system comprising ‘constant companions and casual acquaintances’, which remained above the null rate (individuals associated randomly). The best performing model (lowest QAIC) was “preferred companions + casual acquaintances” (Pref. comp, Casual acqs).

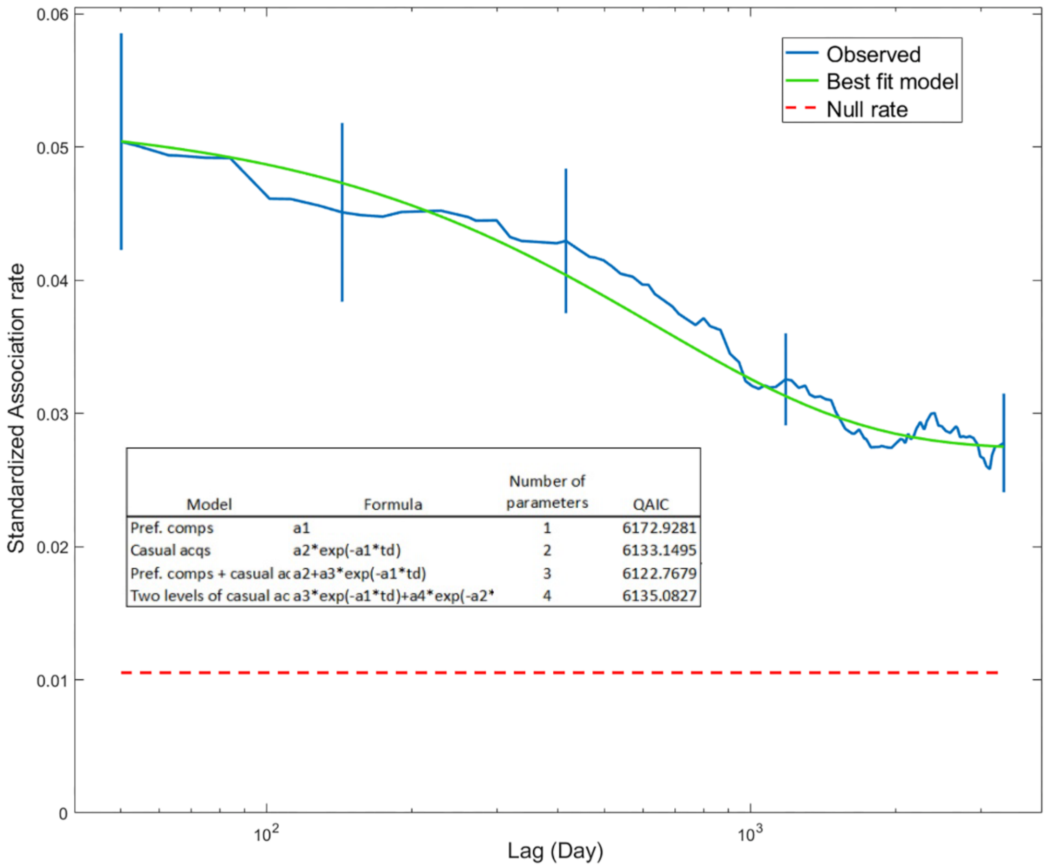

Supplement: S4 Fig — (PDF) [file pone.0270057.s006.pdf]
