# Supplementary material for: Patterns of association and distribution of estuarine-resident common bottlenose dolphins (Tursiops truncatus) in North Carolina, USA
Source: PLoS One. 2022 Aug 15;17(8):e0270057. doi: 10.1371/journal.pone.0270057 (PMC9377618; doi:10.1371/journal.pone.0270057)
Supplement: S1 File — (PDF) [file pone.0270057.s007.pdf]

S1 Doc. Abundance, Potential Biological Removal level, and bycatch of estuarine-resident bottlenose dolphins in North Carolina

S1 Doc for Hohn et al. Patterns of association and distribution of estuarine-resident common bottlenose dolphins (*Tursiops truncatus*) in North Carolina, USA

As with many of the estuarine-resident stocks along the US Atlantic and Gulf of Mexico coasts, the population sizes for the two NC estuarine stocks are small [1]. The NNCESS abundance estimate (upper bound 873; 95% posterior interval = 775-989) [2], from 2013 summer surveys, is thought to be larger than SNCESS. The most recent abundance estimate for SNCESS, from 2006 summer surveys, SNCESS [3] is considered unreliable because it is >8 years old (per US Guidelines for Assessing Marine Mammal Stocks: media.fisheries.noaa.gov/dam-migration/02-204-03.pdf) but was about 25% the size of the NNCESS. The difference in abundance between the two stocks resulted in different annual thresholds for sustainable human-caused mortalities (Potential Biological Removal Level [PBR] under the MMPA) [4]. The PBR for the SNCESS was 1.6 animals annually [5] compared to the still-valid (until 2022) PBR for the NNCESS of 7.8 animals annually [1].

Current abundance estimates and resulting PBR levels for these small stocks in NC are important to determine due to their risk of incidental mortality (i.e., bycatch) or serious injury in commercial fisheries. The NNCESS is known or suspected to interact with 10 NC commercial fisheries operating in NC estuarine and coastal waters and the SNCESS is known or suspected to interact with six commercial fisheries operating in NC estuarine and coastal waters [6-8]. The mid-Atlantic coastal gillnet fishery (hereafter termed coastal gillnets) is the only fishery with systematic federal observer coverage and resulting bycatch estimates [1]. Assigning observed bycaught dolphins to stock, however, is confounded by the four overlapping stocks in coastal waters. Therefore, a maximum mortality is estimated that assumes that all bycatch in a spatiotemporal stratum that could include NNCESS animals belong to NNCESS, resulting in the recent weighted mean annual maximum bycatch in coastal gillnets of 16.42 (CV=0.22) from 2014-2018, which is 210% of PBR. The mortality estimate (2014-2018) for the NNCESS in coastal gillnets was between six and 28 depending on whether none or all observed bycaught dolphins in waters inhabited by the NNCESS were indeed from that stock [9].

The uncertainty around gillnet bycatch mortality of the SNCESS is even more pronounced due to low levels of observer coverage in coastal waters inhabited by this stock. There was no observed bycaught dolphin that could be assigned to the SNCESS during 2007-2015; however, observer coverage was too low to detect a bycatch event [9]. As a result of low levels of (coastal gillnets) or absent (other fisheries) observer coverage, minimum levels of bycatch are known through data from stranded dolphins with evidence such as attached gear or entanglement lesions that indicate an interaction with fisheries [10, 11] or from self-reported takes by commercial fishers. For example, commercial fishers reported that a dolphin was bycaught in their coastal gillnets in 2011 (could have been from SNCESS or the southern coastal migratory stock) and 2015 (SNCESS only) [1]. Based on these fisher reports and nine recovered beach-cast carcasses (strandings) from coastal beaches with lesions suggestive of gillnet entanglement in gillnet gear, for the SNCESS the minimum annual mean bycatch between 2011 and 2015 could have been at least 2.2 animals. This number exceeds the last valid PBR and it represents the lower limit of

bycatch mortality because 1) not all entangled dolphins are reported by fishers, 2) not all dolphins that die, regardless of cause, strand, 3) the location of entanglement or death of stranding is rarely known and 4) the condition of decomposing carcasses impedes the detection of evidence of entanglement if it had occurred [7, 8, 10].

References

1. Hayes SA, Josephson E, Maze-Foley K, Rosel PE, Turek J, Byrd B, et al. US Atlantic and Gulf of Mexico Marine Mammal Stock Assessments 2020. NOAA Technical Memorandum NMFS-NE-271. 2021;Available from: https://media.fisheries.noaa.gov/2021-07/Atlantic%202020%20SARs%20Final.pdf?null%09.
2. Gorgone AM, Eguchi T, Byrd BL, Altman KM, Hohn AA. Estimating the abundance of the northern North Carolina estuarine system stock of common bottlenose dolphins (*Tursiops truncatus*). NOAA Technical Memorandum. 2014;NMFS-SEFSC-664:1-22.
3. Urian KW, Waples DM, Tyson RB, Hodge LE, Read AJ. Abundance of bottlenose dolphins (*Tursiops truncatus*) in estuarine and near-shore waters of North Carolina, USA. Journal of North Carolina Academy of Science. 2014;129(4):165-71.
4. NMFS. Guidelines for preparing stock assessment reports pursuant to the 1994 amendments to the MMPA. National Marine Fisheries Service Instruction 02-204-01. 2016; Available from: https://www.fisheries.noaa.gov/national/marine-mammal-protection/guidelines-assessing-marine-mammal-stocks.
5. Waring GT, Josephson E, Maze-Foley K, Rosel PE. US Atlantic and Gulf of Mexico marine mammal stock assessments--2013. NOAA Tech Memo NMFS NE. 2014;228(464):02543-1026.
6. Byrd BL, Hohn AA. Differential risk of bottlenose dolphin (*Tursiops truncatus*) bycatch in North Carolina, USA. Aquatic Mammals. 2017;43(5):558-69.
7. Peltier H, Dabin W, Daniel P, Van Canneyt O, Dorémus G, Huon M, et al. The significance of stranding data as indicators of cetacean populations at sea: modelling the drift of cetacean carcasses. Ecological Indicators. 2012;18:278-90.
8. Wells RS, Allen JB, Lovewell G, Gorzelany J, Delynn RE, Fauquier DA, et al. Carcass-recovery rates for resident bottlenose dolphins in Sarasota Bay, Florida. Marine Mammal Science. 2015;31(1):355-68.
9. Lyssikatos M, Garrison LP. Common bottlenose dolphin (*Tursiops truncatus*) gillnet bycatch estimates along the US mid-Atlantic Coast, 2007-2015. NEFSC Reference Document 18-07. 2018.
10. Byrd BL, Hohn AA, Lovewell GN, Altman KM, Barco SG, Friedlaender A, et al. Strandings as indicators of marine mammal biodiversity and human interactions off the coast of North Carolina. Fishery Bulletin. 2014;112(1):1-23.
11. Friedlaender AS, McLellan WA, Pabst DA. Characterising an interaction between coastal bottlenose dolphins (*Tursiops truncatus*) and the spot gillnet fishery in southeastern North Carolina, USA. Journal of Cetacean Research Management. 2001;3(3):293-303.
