# Supplementary material for: Patterns of association and distribution of estuarine-resident common bottlenose dolphins (Tursiops truncatus) in North Carolina, USA
Source: PLoS One. 2022 Aug 15;17(8):e0270057. doi: 10.1371/journal.pone.0270057 (PMC9377618; doi:10.1371/journal.pone.0270057)

S2 Doc. Analysis of anomalous sea-surface temperatures as a possible driver for the unexpected distribution of estuarine-resident bottlenose dolphins.

S2 Doc for Hohn et al. Patterns of association and distribution of estuarine-resident common bottlenose dolphins (*Tursiops truncatus*) in North Carolina, USA

During the 2018 mark-recapture abundance survey for the Southern North Carolina Estuarine System Stock (SNCESS) of common bottlenose dolphins, sea-surface temperatures were anomalously cold and the number of individually identified dolphins was much higher than expected. One possibility for the increase in number of dolphins identified was a shift in distribution of estuarine dolphins from more northern estuarine or coastal waters (NNCESS) to the more southern area defined as the winter habitat of SNCESS. We examined whether anomalous SST is likely to have influenced the distribution of dolphins in the survey area relative to the expected habitat for the NNCESS using satellite-derived and in-situ monitors during and in the month preceding the SNCESS surveys.

Methods

Daily, high resolution (1/4° grid) SST and SST anomaly data were obtained from NOAA's Optimum Interpolation Sea Surface Temperature (OISST) which integrates data from satellites, ships, and buoys (https://www.ncei.noaa.gov/data/sea-surface-temperature-optimum-interpolation/v2.1/access/avhrr/) [1, 2]. These data are provided as a grid. We selected points from the grid that were within or nearby the survey area and had corresponding in-situ SST stations nearby. We also selected a grid point in southern Pamlico Sound.

OISST data were validated, where possible, using the in-situ data. For the survey area, in-situ SST data were obtained from the NOAA Center for Operational Oceanographic Products and Services (CO-OPS) (https://tidesandcurrents.noaa.gov/stations.html?type=Physical+Oceanography), NOAA National Data Buoy Center (NDBC) (https://www.ndbc.noaa.gov/), and the Coastal Ocean Research and Monitoring Program (CORM). In-situ water temperatures were compiled for areas near OISST grid points and averaged daily. Fixed-point daily averages were obtained for hourly CO-OPS and CORMP data, and half-hourly NDBC data. SST data collected during the surveys was averaged for the latitude and longitude over which the temperatures were measured as the boats were moving (n=27 to 639 individual measurements per mean temperature). No continuous in-situ SST measurements were available for southern Pamlico Sound.

Results

In-situ observations of SST and OISST estimates were consistent so OISST was considered a reasonable proxy to compare SST in the survey area to SST in Pamlico Sound. SST was cooler than the long-term average throughout the nearshore and estuarine waters during December and early January (Fig 1), with the anomaly greater (cooler) in the survey area than in Pamlico Sound (Fig 2). While the SST anomaly remained greater (cooler) in the survey area during the survey, it was small and approached zero in southern Pamlico Sound after the first week of the survey.

Discussion

While cooler water temperatures may have resulted in increased movement of estuarine-resident dolphins to more southern coastal waters initially, the anomalous SST in both areas and for a longer period of time in the southern area may not support SST as the driver for the occurrence of dolphins presently assigned to the NNCESS identified in the winter range of SNCESS.

References

1. Banzon V, Smith T, Steele M, Huang B, Zhang H-M. Improved estimation of proxy sea surface temperature in the arctic. Journal of Atmospheric and Oceanic Technology. 2020;37(2):341-9.
2. Huang B, Liu C, Banzon V, Freeman E, Graham G, Hankins T, et al. Improvements of the daily optimum interpolation sea surface temperature (DOISST) version 2.1. Climate. 2021;34:2923-39.

Fig 1. Sea-surface temperature (SST) and SST anomaly in southern North Carolina estuarine and coastal waters prior to and during the survey.

SST and SST anomalies in southern North Carolina in the months prior to (Dec 2017) and during (Jan 2018) (vertical dashed lines) the mark-recapture survey in the defined winter range of the SNCESS. (A) Solid lines show daily SST from NOAA's OISST. SST in the area of the survey (solid purple line) was validated using *in-situ* continuous SST collected by NOAA CO-OPS (purple dashed line). SST data collected during the survey are shown as gray diamonds (SST in the estuary) and black circles (SST on the coast). No continuous sampling station was available for southeastern Pamlico Sound. (B) Sea-surface temperature (SST) anomalies. The pink and purple lines show the anomalies in the northern and southern areas of the current survey. The green line shows the SST anomaly in southern Pamlico Sound. Gaps indicate missing data.

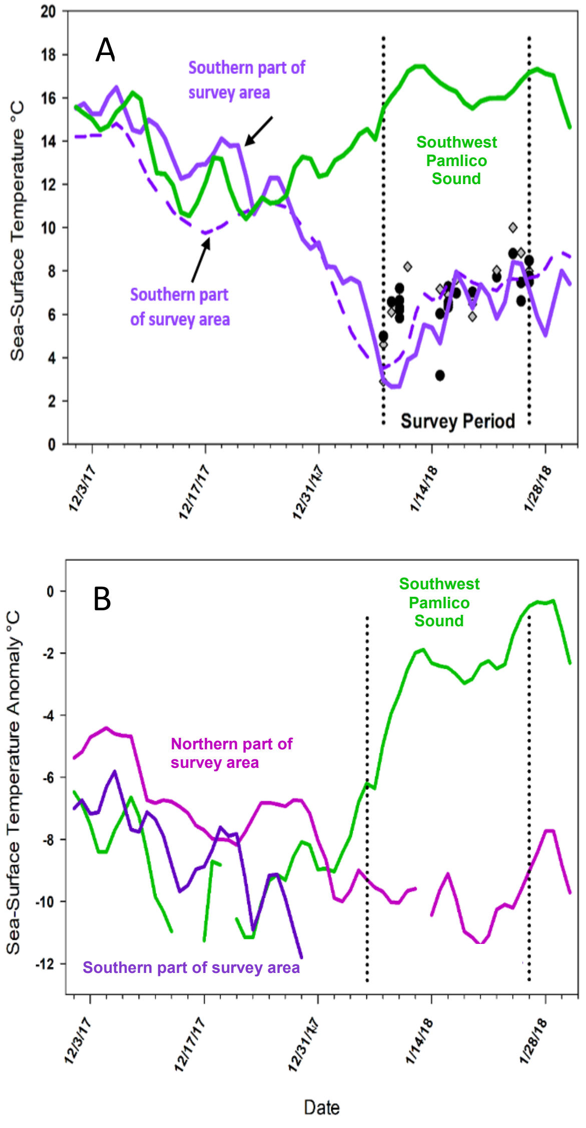

Supplement: S2 File — (PDF) [file pone.0270057.s008.pdf]
