# Supplementary material for: Patterns of association and distribution of estuarine-resident common bottlenose dolphins (Tursiops truncatus) in North Carolina, USA
Source: PLoS One. 2022 Aug 15;17(8):e0270057. doi: 10.1371/journal.pone.0270057 (PMC9377618; doi:10.1371/journal.pone.0270057)
Supplement: S3 File — (PDF) [file pone.0270057.s009.pdf]

S3 Doc. Criteria for images and examples of dorsal fins with long-term marks (LTM) and short-term marks (STM).

S3 Doc for Hohn et al. Patterns of association and distribution of estuarine-resident common bottlenose dolphins (*Tursiops truncatus*) in North Carolina, USA

A combination of distinctiveness and photo quality determines whether a photograph can be used for photo-identification using long-term marks (LTM) or short-term marks (STM). STM are thought to be useful at least within a survey and may result in a larger sample size for analyses.

Criteria for Identifying LTM	Criteria for Identifying STM
Very distinctive/distinctive and photo quality excellent or good	Very distinctive/distinctive and photo quality fair
Slightly distinctive and photo quality excellent	Slightly distinctive and photo quality good or fair
	Not distinctive with small notches scratches/scars and photo quality excellent

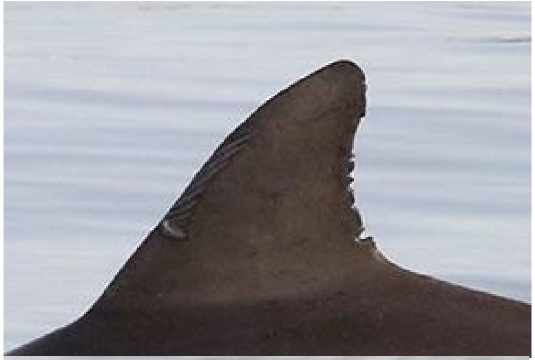

A. Excellent photo quality and highly distinctive fin with LTM.

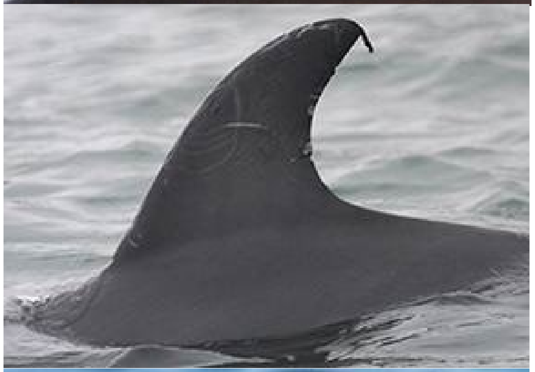

B. Excellent photo quality and indistinctive fin with STM.

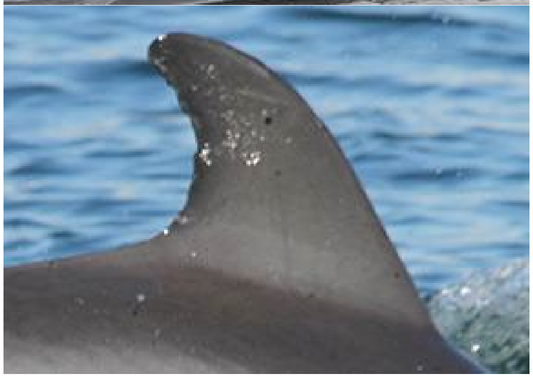

C. Poor photo quality and slightly distinctive fin - not usable.
