# Supplementary material for: Patterns of association and distribution of estuarine-resident common bottlenose dolphins (Tursiops truncatus) in North Carolina, USA
Source: PLoS One. 2022 Aug 15;17(8):e0270057. doi: 10.1371/journal.pone.0270057 (PMC9377618; doi:10.1371/journal.pone.0270057)

S4 Doc. Prevalence of *Xenobalanus globicipitis* on estuarine-resident common bottlenose dolphins in NC

S4 Doc for Hohn et al. Patterns of association and distribution of estuarine-resident common bottlenose dolphins (*Tursiops truncatus*) in North Carolina, USA.

It has been suggested that the prevalence of the monospecific commensal barnacle, *Xenobalanus globicipitis*, [1] may be used to distinguish between sympatric coastal and estuarine-resident dolphins [2-4]. To determine whether the prevalence of *Xenobalanus* might help define stocks or clusters, we test for differences in infestation among groups by habitat, within and among individuals over time, and among clusters.

Methods

First, we examined the prevalence of *Xenobalanus* in estuarine-resident dolphin groups (referred to as load) sighted during the 2018 survey, comparing groups sighted on the coast with those sighted in the estuary (Chi-squared, SAS Proc FREQ). Sightings of groups presumed to be part of the coastal migratory stock had previously been identified, in part from heavy infestations, and were excluded from these analyses.

Second, after the social network analysis identified the 95 individuals in the clusters, we counted *Xenobalanus* on the dorsal-fin for each sighting of each individual included in the clusters using the photo-id images in the long-term 2018 SNCESS and NOAA Beaufort Catalogs. The counts were then stratified into categories (none, light 1-5, medium 6-10, heavy >10) [5] for analyses; despite the categorization, we refer to these data as counts to prevent ambiguity with use of load, which we will restrict to group characteristics. Same-day sightings were excluded as no change in *Xenobalanus* counts would be expected within the same day. We summarized the count categories for each individual across all sightings as (a) seen with the same level of infestation and the category of that level, (b) seen with both none and a light load, and (c) at least one sighting was with a medium or heavy load. The latter two strata also indicated that there was a change in counts across sightings. Differences in the summarized categories among clusters were tested with a Chi-squared test (SAS Proc FREQ). The α level used for statistical significance was 0.05.

Differences in *Xenobalanus* counts for each sighting of each individual were tested using a multinomial mixed model with individual dolphin as a random effect to control for non-independence among the repeated observations for each individual (SAS Proc GLIMMIX). Possible covariates included cluster, habitat, and season. We also tested the possible covariates *Xenobalanus* count, season, habitat, and stock stratum on cluster membership using the multinomial mixed model. For the two sightings in SC, we considered the stock stratum to be SNCESS (SC is not a defined stock stratum); neither individual had a sighting history in unique NNCESS strata so this assignment seemed reasonable.

Results

Xenobalanus load varied among groups by habitat of sighting. The infestation was present on dolphins in 39 of the 88 groups sighted on-effort during the 2018 survey. Significantly more coastal groups (32 of 53, 60%) had an infestation than estuarine groups (seven of 35, 20%) (Chi-squared, $p<0.001$) (Table 1).

Counts of *Xenobalanus* on individuals in the six social clusters resulted in 703 sightings with none, 131 with a light, 16 with a medium, and 15 with a heavy infestation (Table 2), excluding 59 same-day sightings and 19 sightings for which counts were not available. By individual (n=95 individuals in the six clusters), *Xenobalanus* counts were constant across all sightings for only 33, all with no *Xenobalanus* (from 7-17 sightings) (Table 3). Changes in *Xenobalanus* counts varied between none and light for 44 individuals. For the remaining 18 individuals, found in four clusters (A-C, F), there was least one sighting with a medium or heavy count. Albeit with small samples sizes, medium and heavy counts occurred in the summer only in Clusters A and B (3 of 5 and 9 of 12 sightings in summer, respectively) (Fig 1). In contrast, all medium and heavy counts occurred only in winter and spring in Clusters C (12 of 12 sightings in winter and spring) and F (2 of 2). Only one individual (Cluster F), one of two with ≥7 sightings seen in SC coastal waters during the 2018 survey, was always seen with *Xenobalanus*, mostly with a light load (n=10 total sightings from 2003 to 2018).

For the three large clusters (Clusters A-C) (n=90 individuals), differences occurred in *Xenobalanus* counts among clusters (Table 3). Relative to the other two clusters, Cluster A had fewer individuals with *Xenobalanus* (Chi-squared, $p=0.011$) and more individuals never seen with *Xenobalanus* and, thus, for which there was no change in number of *Xenobalanus* (Chi squared, $p<0.001$). No difference was detected between Cluster B and Cluster C in these characteristics.

Across all sightings of each individual for Clusters A-C (n=825 sightings), habitat and season were significant covariates for *Xenobalanus* counts (multinomial mixed model, $p=0.003$ and $p<0.001$, respectively), with *Xenobalanus* significantly more prevalent in coastal sightings and during winter and spring. Cluster was not a significant predictor overall ($p=0.070$), but pairwise contrasts showed significantly more *Xenobalanus* in Cluster C relative to Cluster A ($p=0.031$) and marginally significantly more in Cluster B relative to Cluster A ($p=0.051$).

Discussion

Differences in *Xenobalanus* loads and counts differed between clusters. While the conditions required for infestation of *Xenobalanus* are unknown, the current results, showing *Xenobalanus* loads in groups and on individuals being lower in estuarine than coastal waters, supports that salinity may be important in determining presence and abundance of *Xenobalanus* on dorsal fins. In estuarine waters of North Carolina, salinity is inversely related to distance from ocean inlets due to river input in the west, where average salinity in winter is 6-8 ppt [6, 7]. While salinity in coastal areas covered during the current survey was greater than that in estuarine waters, the difference was relatively small (average of 33 ppt vs 28 ppt), reflecting the difference in estuarine habitat and possibly accounting for year-round *Xenobalanus* infestation in the Beaufort

and southern NC clusters. Along the northern South Carolina coasts and estuaries, Silva et al. [8] found *Xenobalanus* to be more prevalent on presumed estuarine-stock groups seen on the coast than in the estuary. In the current study, it was somewhat surprising that the Southern NC community had the lowest infestation rather than the Pamlico Sound community, the latter of which appears to spend more time in lower-salinity estuarine waters. However, only the coastally oriented communities had medium or high *Xenobalanus* loads during the summer and members of the Pamlico Sound community carried more *Xenobalanus* in the winter and spring when sightings were all coastal or within the estuarine system near the coast. These results may also reflect seasonally uneven sampling.

Xenobalanus loads are not static. During a 3-yr photo-id survey along the coast of New Jersey, Toth-Brown and Hohn [5] documented changes, albeit small, in the number and size of presence/absence of *Xenobalanus*, there were changes in position on the fin and size indicating between-year replacement. There was also a seasonal effect (five dolphins with no infestation from May-August had an average of 4-5 small *Xenobalanus* in September) and year effect (some individuals had no infestation in some years and had a light to medium load in others). Silva et al. [8] reported no change in relative *Xenobalanus* load between warm and cool months on presumed estuarine groups, and an increase in load during winter for coastal groups, albeit with small sample sizes. Results from the current survey also showed significantly more *Xenobalanus* in winter and spring relative to summer and fall in estuarine-resident dolphins. However, across the time frame of our long-term photo-id catalog, our effort has primarily been in the summer during targeted, short-term surveys. Year-round effort across the distribution of the estuarine resident communities may lead to a better understanding of *Xenobalanus* infestation in the estuarine and near-shore coastal waters of NC.

Uncertainty in interpreting *Xenobalanus* loads pertains to a lack of knowledge on timing or causes of settlement and shedding on dorsal fins. Proposed mechanisms include dolphin age or health, or environmental conditions such as water temperature, upwelling conditions, or long-term environmental changes [5, 9-12]. Seasonal changes in load have been proposed to represent the life-cycle or temperature or nutrient sensitivity [11, 12]. It has been suggested, for example, that migratory killer whales (Orcinus orca) sighted with *Xenobalanus* in the Arctic acquired the infestation while in warmer waters [13]. *Xenobalanus* has a cosmopolitan distribution from polar to tropical waters [1, 13, 14] and, thus, would be expected to be tolerant of seasonal water temperatures in NC. Dreyer et al. [15] suggest that attachment occurs during coastal aggregations of cetacean hosts following fast (8 day) larval development. Our long-term sighting results, as well as sightings during the current survey, confirming that dolphins move between estuarine and coastal waters, provide an opportunity for coastal infestation. The current findings may lead to more research on the potential use of *Xenobalanus* as biological tags for stock membership [1, 9, 16], although season may have to be considered.

References

1. Kane EA, Olson PA, Gerrodette T, Fiedler PC. Prevalence of the commensal barnacle *Xenobalanus globicipitis* on cetacean species in the eastern tropical Pacific Ocean, and a review of global occurrence. Fishery Bulletin. 2008;106(4):395-404.
2. Griffin EK, Rosel PE, Balmer BC, Perrtree RM, Cox TM. Using photo-identification and genetic data to examine fine-scale population structure of common bottlenose dolphins (*Tursiops truncatus*) in the estuarine waters surrounding Savannah, Georgia. Aquatic Mammals. 2021;47(3).
3. Urian KW, Kaufmann R, Waples DM, Read AJ. The prevalence of ectoparasitic barnacles discriminates stocks of Atlantic common bottlenose dolphins (*Tursiops truncatus*) at risk of entanglement in coastal gill net fisheries. Marine Mammal Science. 2019;35(1):290-9.
4. Byrd B, Eguchi T, Gorgone A, Toth JL, Hohn A, A. *Xenobalanus* isn't just a drag: Can the *presense* of Xenobalanus help differentiation stocks of bottlenose dolphins along the U.S. Atlantic coast? 19th Bienniel Conference on the Biology of Marine Mammals 2011.
5. Toth-Brown J, Hohn A. Occurrence of the barnacle, *Xenobalanus globicipitis*, on coastal bottlenose dolphins (*Tursiops truncatus*) in New Jersey. Crustaceana-International Journal of Crustacean Research. 2007;80(10):1271-9.
6. Giese GL, Wilder HB, Parker Jr GG. Hydrology of major estuaries and sounds of North Carolina. Report. 1985. Report No.: 2221.
7. Epperly SP, Ross SW. Characterization of the North Carolina Pamlico-Albemarle estuarine complex. NOAA Technical Memorandum. 1986;NMFS-SEFSC-175:1-55.
8. Silva D, Young R, Lavin A, O'Shea C, Murray E. Abundance and seasonal distribution of the Southern North Carolina estuarine system stock (USA) of common bottlenose dolphins (*Tursiops truncatus*). Journal of Cetacean Research and Management. 2020;21(1):33-43.
9. Aznar FJ, Míguez-Lozano R, Ruiz B, de Castro AB, Raga J, Blanco C. Long-term changes (1990-2012) in the diet of striped dolphins *Stenella coeruleoalba* from the western Mediterranean. Marine Ecology Progress Series. 2017;568:231-47.
10. Siciliano S, Cardoso J, Francisco A, De Souza SP, Hauser-Davis RA, Iwasa-Arai T. Epizoic barnacle (*Xenobalanus globicipitis*) infestations in several cetacean species in south-eastern Brazil. Marine Biology Research. 2020;16(5):356-68.
11. Orams MB, Schuetze C. Seasonal and age/size-related occurrence of a barnacle (*Xenobalanus globicipitis*) on bottlenose dolphins (*Tursiops truncatus*). Marine Mammal Science. 1998;14(1):186-9.
12. Van Waerebeek K, Reyes J, Aflaro J. Helminth parasites and phoronts of dusky dolphins *Lagenorhychus obscurus* (Gray, 1828) from Peru. Aquatic Mammals. 1993;19(3):159-69.
13. Matthews CJD, Ghazal M, Lefort KJ, Inuarak E. Epizoic barnacles on Arctic killer whales indicate residency in warm waters. Marine Mammal Science. 2020;36(3):1010-4.
14. Ólafsdóttir D, Shinn AP. Epibiotic macrofauna on common minke whales, *Balaenoptera acutorostrata* Lacépède, 1804, in Icelandic waters. Parasites & Vectors. 2013;6(1):105.
15. Dreyer N, Zardus JD, Høeg JT, Olesen J, Yu M-C, Chan BKK. How whale and dolphin barnacles attach to their hosts and the paradox of remarkably versatile attachment structures in cypris larvae. Organisms Diversity & Evolution. 2020;20(2):233-49.
16. Whitehead TO, Rollinson DP, Reisinger RR. Pseudostalked barnacles *Xenobalanus globicipitis* attached to killer whales *Orcinus orca* in South African waters. Marine Biodiversity. 2015;45(4):873-6.

Table 1. Prevalence of *Xenobalanus* on dolphin groups by habitat of sighting.

Prevalence of *Xenobalanus globicipitis* for 88 groups of estuarine-resident common bottlenose dolphins seen on-effort during the 2018 winter survey. Percent *Xenobalanus* is the percentage of individual dolphins in a group with *Xenobalanus*. When present, the infestation load was categorized as light, medium, or heavy. By definition, groups with >70% prevalence of *Xenobalanus* and an infestation load of medium or heavy and were difficult to approach were presumed to be coastal migratory stock and are excluded, with the exception that one group with a medium load sighted in the surf zone and approachable for photography was included as a presumed estuarine animal.

Percent *Xenobalanus*	*Xenobalanus* Load				
	None	Light	Medium	Heavy	Total
**Coast**					
0%	21				21
1-25%		16	1		17
26-50%		8	2		10
51-75%		1			1
76-100%		3	1		4
**Estuary**					
0%	28				28
1-25%		7			7
26-50%					
51-75%					
76-100%					
**Total**	49	35	4		88

Table 2. Prevalence of *Xenobalanus* on dolphins by cluster.

Counts of *Xenobalanus* on dorsal fins across all sightings of each individual in the long-term NOAA Beaufort photo-id catalog for the 95 estuarine-resident common bottlenose dolphins that were included in the six clusters (A through F) from the social network analysis. The counts were stratified by sighting location, estuary or coast, and then categorized as None (no infestation), Light (1-5), Medium (5-10), or Heavy (>10). Same-day sightings (n=59) were excluded and counts were unavailable for 19 photographs.

Cluster	Habitat of Sighting	Number of Dolphins	Total Sightings	*Xenobalanus* counts			
				None	Light	Medium	Heavy
A	Estuary	23	189	170	16	0	3
	Coast		29	22	5	2	0
B	Estuary	32	254	204	42	4	4
	Coast		37	26	7	2	2
C	Estuary	35	304	254	40	4	6
	Coast		12	2	8	2	0
D	Estuary	3	20	17	3	0	0
	Coast		3	1	2	0	0
E	Estuary	1	7	7	0	0	0
	Coast		0	0	0	0	0
F	Estuary	1	9	0	8	1	0
	Coast		1	0	0	1	0
Total		95	865	703	131	16	15

Table 3. Changes in *Xenobalanus* counts on dorsal fins of the 90 dolphins included in the three large clusters.

Changes in *Xenobalanus* counts on dorsal fins of all sightings of 90 estuarine-resident bottlenose dolphins included in the three larger clusters identified as part of the current study. No change indicates that the load did not change across all sightings for an individual, change between none and a light load indicates that an individual was seen in both states, change from a lighter load to medium or heavy indicates that individual was seen with a medium or heavy load as well as with none or a light load.

Cluster	No Change (all None)	Change Between None or Light	Change from Lighter Load to Medium or Heavy	Total Number of Individual Dolphins
A	15	5	3	23
B	6	20	6	32
C	11	16	8	35
Total	32	41	17	90

Fig 1. Relative levels of *Xenobalanus* on individuals by cluster and habitat.

The proportion of sightings of estuarine-resident bottlenose dolphins with the four categories of *Xenobalanus* counts. Sightings are stratified by habitat and cluster, for the three clusters with >3 individuals (A-C), so each cluster/habitat category sums to 1.0. Clusters were determined from a social network analysis. Count category was determined by counts of *Xenobalanus* on the dorsal fin for each sighting of each individual, excluding same-day sightings, then categorized as None (no infestation), Light (1-5), Medium (5-10), or Heavy (>10) (53)). The numbers above the bars is the sample size for each group, *e.g.*, for Cluster A there were 190 estuarine sightings from which *Xenobalanus* were counted.

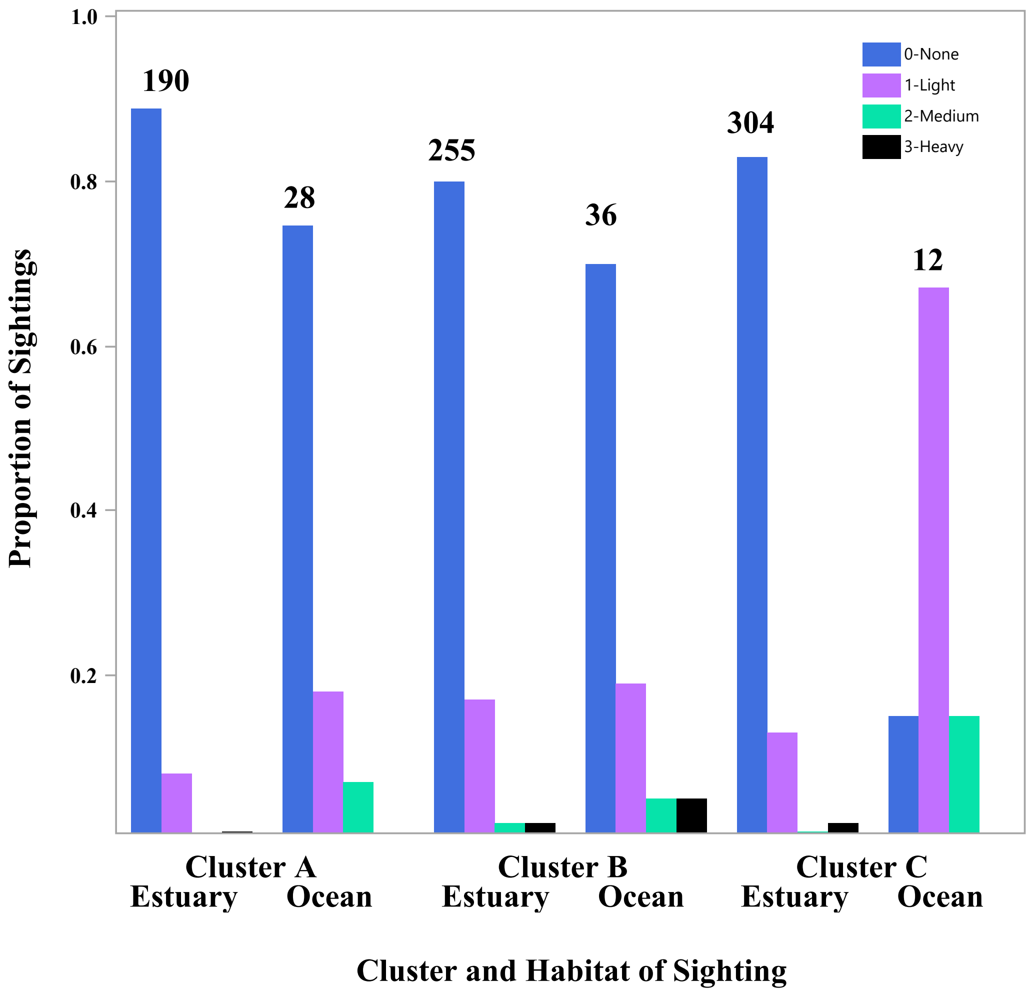

Supplement: S4 File — (PDF) [file pone.0270057.s010.pdf]
